# Trem2 deficiency impairs recovery and phagocytosis and dysregulates myeloid gene expression during virus-induced demyelination

**DOI:** 10.1186/s12974-022-02629-1

**Published:** 2022-11-04

**Authors:** Mihyun Hwang, Carine Savarin, Jihye Kim, Jennifer Powers, Natasha Towne, Hyunsuk Oh, Cornelia C. Bergmann

**Affiliations:** 1 Department of Neurosciences, Lerner Research Institute, Cleveland Clinic Foundation, Cleveland, OH USA; 2 Department of Quantitative Health Sciences, Lerner Research Institute, Cleveland Clinic Foundation, Cleveland, OH USA; 3 Center for Immunotherapy and Precision Immuno-Oncology, Lerner Research Institute, Cleveland Clinic Foundation, Cleveland, OH USA

**Keywords:** TREM2, CNS, Virus, Demyelination, Microglia, BMDM, Phagocytosis, Coronavirus

## Abstract

**Background:**

Triggering receptor expressed on myeloid cells 2 (Trem2) plays a protective role in neurodegenerative diseases. By contrast, Trem2 functions can exacerbate tissue damage during respiratory viral or liver infections. We, therefore, investigated the role of Trem2 in a viral encephalomyelitis model associated with prominent Th1 mediated antiviral immunity leading to demyelination.

**Methods:**

Wild-type (WT) and Trem2 deficient (*Trem2*^−/−^) mice were infected with a sublethal glia tropic murine coronavirus (MHV–JHM) intracranially. Disease progression and survival were monitored daily. Leukocyte accumulation and pathological features including demyelination and axonal damage in spinal cords (SC) were determined by flow cytometry and tissue section immunofluorescence analysis. Expression of select inflammatory cytokines and chemokines was measured by RT-PCR and global myeloid cell gene expression in SC-derived microglia and infiltrated bone-marrow-derived macrophages (BMDM) were determined using the Nanostring nCounter platform.

**Results:**

BMDM recruited to SCs in response to infection highly upregulated *Trem2* mRNA compared to microglia coincident with viral control. Trem2 deficiency did not alter disease onset or severity, but impaired clinical recovery after onset of demyelination. Disease progression in *Trem2*^−/−^ mice could not be attributed to altered virus control or an elevated proinflammatory response. A prominent difference was increased degenerated myelin not associated with the myeloid cell markers IBA1 and/or CD68. Gene expression profiles of SC-derived microglia and BMDM further revealed that Trem2 deficiency resulted in impaired upregulation of phagocytosis associated genes *Lpl* and *Cd36* in microglia, but a more complex pattern in BMDM.

**Conclusions:**

Trem2 deficiency during viral-induced demyelination dysregulates expression of other select genes regulating phagocytic pathways and lipid metabolism, with distinct effects on microglia and BMDM. The ultimate failure to remove damaged myelin is reminiscent of toxin or autoimmune cell-induced demyelination models and supports that Trem2 function is regulated by sensing tissue damage including a dysregulated lipid environment in very distinct inflammatory environments.

**Supplementary Information:**

The online version contains supplementary material available at 10.1186/s12974-022-02629-1.

## Introduction

Trem2 is an immunoreceptor belonging to the TREM family that is constitutively expressed in tissue macrophages, microglia, and osteoclast precursors [1, 2]. It has received considerable attention based on the linkage of TREM2 variants and dysfunction to a range of neurodegenerative diseases, including Nasu-Hakola and Alzheimer’s disease (AD) [2–4]. Trem2 is known to bind to numerous ligands, among them phospholipids, glycolipids and apolipoproteins, as well as phagocytose apoptotic cells and cellular debris upon tissue damage [5]. In addition to regulating phagocytosis, lipid metabolism, and inflammatory factors, Trem2 also can promote myeloid cell expansion, differentiation, survival and myeloid chemotaxis in response to inflammation or cell damage [1–5]. In animal models of demyelinating disease induced by Cuprizone (CPZ) or autoimmune-T cells, Trem2 is required for uptake and degradation of myelin debris in demyelinated lesions, thereby facilitating remyelination [6]. Loss of Trem2 results in defective myelin debris clearance coincident with decreased microglia activation [7–9], while activation of Trem2 induces increased phagocytic and lysosomal activity [10, 11]. Moreover, the transfer of Trem2 expressing myeloid cells in the experimental allergic encephalomyelitis (EAE) model of multiple sclerosis (MS) promotes removal of damaged myelin and an anti-inflammatory milieu [11]. These studies support that Trem2 activation on both microglia and central nervous system infiltrating BMDM can ameliorate the accumulation of damaged myelin and thereby promote remyelination.

Trem2 is also associated with macrophage activation during virus and bacterial infections in distinct organs, namely, the liver or lung [12–18]. A context-specific role for Trem2 in regulating virus replication as well as pathogenesis is highlighted by its protective but also detrimental effects. For example, Trem2 facilitates macrophage infection by porcine reproductive and respiratory syndrome virus (PRRSV) and suppresses a protective proinflammatory response [13]. By contrast, Trem2 aggravates disease in both lymphocytic choriomeningitis virus (LCMV) infection of the liver and Sendai virus infection of the lung, albeit involving very distinct mechanisms [12, 15]. Despite similar T cell responses, Trem2 deficiency accelerated LCMV virus clearance and ameliorated liver damage [15]. By contrast, during Sendai virus infection Trem2 deficiency did not affect viral control, but prevented development of chronic inflammatory respiratory disease driven by soluble Trem2 (sTrem2) mediated enhanced macrophage survival [12]. Integration of various Trem2 functions in different contexts can thus determine distinct disease outcomes.

The potential impact of Trem2 deficiency on both virus control and proinflammatory functions, in addition to phagocytosis, thus prompted us to investigate the role of Trem2 in viral pathogenesis and associated demyelination induced by a neurotropic, attenuated coronavirus mouse hepatitis virus (MHV) designated MHV–JHM. Infection with glial tropic MHV–JHM causes acute encephalomyelitis manifested by recruitment of leukocytes, including BMDM, neutrophils and T cells. A highly polarized Th1 response comprising IFNγ production and cytolytic activity controls infectious virus, but in the process also induces demyelination [19–21]. Efforts to delineate the role of microglia and infiltrating BMDM in this model reveal a complex picture. Demyelination can occur in the absence of CNS infiltrating BMDM [22, 23]. However, drug-induced microglia ablation exacerbates demyelination and impairs remyelination [24]. Finally, overexpression of the monocyte recruiting chemokine CCL2 induces myelin loss in Rag^−/−^ mice which otherwise show sparse demyelination [25]. Taken together these studies suggest that both microglia and BMDM can participate in removal of myelin from axons, but microglia are essential for remyelination. This may reside in a more efficient machinery to remove and degrade damaged myelin in addition to production of other disease resolving factors. Demyelination in the virus model is distinct from other studies of Trem2 function, which rely on direct induction of oligodendrocyte death or autoimmune attack [7, 8, 10, 11]. Comparison of pathogenesis in MHV–JHM-infected *Trem2*^−/−^ and WT mice revealed that the absence of Trem2 had no impact on virus control or overall immune responses in spinal cords. However, ongoing clinical disease in *Trem2*^−/−^ mice was associated with the inability to remove damaged myelin as well as selectively impaired gene expression of factors related to phagocytosis in microglia as well as in BMDM. Our data thus demonstrate that Trem2 does not impact antiviral immune responses in the CNS, but is essential for disease resolution by assuring clearance of myelin debris.

## Materials and methods

### Mice, virus, and infection

*Trem2*^−/−^ mice (Trem2tm1(KOMP)Vlcg) in which exons 2–4 of the *Trem2* gene were replaced with *LacZ* resulting in loss of Trem2 function, were generated by the trans-NIH Knock-Out Mouse Project (KOMP) and kindly provided by Dr. Bruce T Lamb (Indiana University School of Medicine, Indianapolis, IN) [26]. *Trem2*^*−/−*^ mice were housed under specific pathogen-free conditions at an accredited barrier facility at the Cleveland Clinic Lerner Research Institute. C57BL/6J (Stock #000664) mice were purchased from the Jackson Laboratory (Bar Harbor, ME). Mice of both sex were infected intracranially (i.c.) at 6 weeks of age with 2000 plaque forming units (PFU) of the glia tropic monoclonal antibody (mAb)-derived 2.2v-1 variant of MHV strain JHM (MHV–JHM) [27]. Clinical disease was scored daily as described [28]: 0, healthy; 1, ruffled fur and hunched back; 2, hind limb paralysis/inability to turn to upright position; 3, complete hind limb paralysis and wasting; 4, moribund/dead. All animal procedures were approved by the Institutional Animal Care and Use Committee of the Cleveland Clinic (PHS assurance number A3047-01) and were conducted in compliance with the Guide for the Care and Use of Laboratory Animals from the National Research Council.

### Isolation of CNS cells and flow cytometric analysis

SCs from mice perfused with cold phosphate buffered saline (PBS) were homogenized in Dulbecco’s phosphate-buffered saline (DPBS, pH 7.4) using Tenbroeck tissue homogenizers as described [28]. Homogenates were centrifuged at 450×*g* for 10 min at 4 °C. Cells were re-suspended in RPMI containing 25 mM HEPES (pH7.2), adjusted to 30% Percoll (Pharmacia, Uppsala, Sweden), underlaid with 1 ml 70% Percoll and centrifuged at 850×*g* for 30 min at 4 °C. Cells were collected from the 30%/70% interface, washed with RPMI, counted and suspended in fluorescent activated cell sorting (FACS) buffer (0.1% Bovine serum albumin in DPBS). Fcγ receptors were blocked with 1% mouse serum and rat anti-mouse CD16/32 mAb (clone 2.4G2: BD Biosciences, San Diego, CA) for 20 min on ice prior to staining with fluorescein isothiocyanate (FITC)-, phycoerythrin (PE)-, peridinin chlorophyll protein (PerCP)-, or allophycocyanin (APC)-conjugated mAbs specific for CD45 (clone 30-F11), CD8 (clone 53-6.7), CD4 (clone GK1.5), Ly6G (clone 1A8), CD11b (clone M1/70), MHC class II (clone M5/114.15.2) (all from BD Bioscience, Mountain View, CA) in FACS buffer. Virus specific CD8 T cells specific for the Spike protein peptide S510 were detected with PE-conjugated Db/S510 Class I tetramer (iTAg™ MHC Class I tetramer, Beckman Coulter Immunomics, San Diego, CA) at 0.1 μg/0.5–1.0 × 10^6^ cells as directed by the supplier [29]. Samples were analyzed on a BD ACCURI™ C6 PLUS (BD Biosciences). Forward and side scatter signals obtained in linear mode were used to establish a gate containing live cells while excluding dead cells and tissue debris. Data were analyzed using FlowJo™ v10.7.1 software (BD Life Sciences, Ashland, OR).

### Cell sorting and nCounter analysis

For microglia and BMDM isolation, 5–6 SCs from PBS-perfused WT and *Trem2*^−/−^ mice were finely minced with a razor blade individually. Minced tissues were enzymatically digested using the papain-based Neural Tissue Dissociation Kit (P) according to the manufacture’s instruction (Miltenyi Biotec). After digestion, SC-derived cells were isolated using percoll gradients as described above, and then stained with CD45, CD11b and Ly6G for 30 min on ice. SC-derived microglia (CD45^int^CD11b^+^) and BMDM (CD45^hi^CD11b^+^Ly6G^−^) were purified using a FACSAria cell sorter (BD Biosciences) using a 85 m nozzle (Additional file 4: Fig. S4). Microglia from naïve mice and circulating monocytes were used as controls for microglia and SC-infiltrated BMDM after infection, respectively. Myeloid cell yields from individual SC ranged from 40,000 to 150,000 cells. The minimum cell number used for RNA extraction was 80,000 cells; cells from 2 SCs were pooled, when yields per cord were below 50,000. RNA was prepared by extraction with TRIzol reagent (Invitrogen, Carlsbad, CA, USA) and RNA Clean and Concentrator™-5 (Zymo Research, Irvine, CA, USA) according to the manufacturer’s instructions. 100 ng of RNA was applied for the Nanostring analysis. The NanoString nCounter system directly captures and counts individual mRNA transcripts using a multiplexed measurement system thereby omitting cDNA-based amplification (34). Seven hundred sixty-four transcripts were qualified with the NanoString nCounter Myeloid Innate Immune Panel according to the manufacturer’s protocol (NanoString Technologies, Seattle, WA, USA). ROSALIND^®^ follows the nCounter^®^ Advanced Analysis protocol of dividing counts within a lane by the geometric mean of the normalizer probes from the same lane. Housekeeping probes used for normalization are selected based on the geNorm algorithm in the NormqPCR R library. Normalized count value were based on housekeeping genes included as a reference in the nCounter gene panels. ROSALIND performs a filtering of Cell Type Profiling results to include results that have scores with a *p* value greater than or equal to 0.05. Enrichr [30] with PanglaoDB [31] and MSigDB Hallmark [32] was used to confirm the major cell types of samples. R (version 4.1) (https://www.R-project.org/) was used to produce additional volcano plots and heatmaps of comparisons.

### Gene expression analysis by RT-PCR

RNA from individual SCs, brains and cervical lymph nodes was extracted using TRIzol reagent (Invitrogen, Carlsbad, CA) according to the manufacturer’s instructions. Following treatment with DNase I using DNA free kit (Ambion, Austin, TX), cDNA was synthesized using Moloney Murine Leukemia Virus reverse transcriptase (Invitrogen) in buffer containing 10 mM deoxynucleoside triphosphate mix, 250 ng random hexamer primers and oligo (dT) (1:1 ratio) (Invitrogen). RNA expression was assessed using either SYBR Green master mix (Applied Biosystems, Foster city, CA) or Taqman fast master mix (Applied Biosystems, Foster City, CA) as described [33]. The following primers were used with SYBR Green master mix: *Gapdh*, F, 5′-CATGGCCTTCCGTGTTCCTA-3′, and R, 5′-ATGCCTGCTTCACCACCTTCT-3′; and *Viral-Nucleocapsid (N)*, F, 5′-CGCAGAGTATGGCGACGAT-3′, and R, 5′-GAGGTCCTAGTCTCGGCCTGTT-3′; *Tnf, F, 5*′*-*GCCACCACGCTCTTCTGTCT*-3*′*,* and R, 5′-GGTCTGGGCCATAGAACTGATG-3′; *iNOS* F, 5′-GTT CTC AGC CCA ACA ATA CAA GA-3′, and R, 5′-GTG GAC GGG TCG ATG TCA C-3′; *Arginase1*, F, 5′-TGGGTGGATGCTCACACTGA-3′, and R, 5′-CAGGTTGCCCATGCAGATT-3′; *Tgfb*, F, 5′-CCCGAAGCGGACTACTATGC-3′, and R, 5′-CGAATGTCTGACGTATTGAAGAACA-3′. Taqman fast master mix and Taqman primers/probes were used to assess *Gapdh, Trem2, Tyrobp, Ccl2* and *Ifnγ*. mRNA levels were determined using the 7500 Fast Real-Time PCR System (Applied Biosystems). Gene expression was normalized *to Gapdh* expression and converted to a linearized value using the formula: 2^(Ct GAPDH-Ct gene) × 1000.

### Histology

SCs were collected following trans-cardiac perfusion of infected mice with PBS followed by 4% paraformaldehyde (PFA) in PBS, post-fixed overnight, and cryopreserved in 30% sucrose. SCs were cut into six to seven pieces and embedded in TissueTeck OCT Compound embedding medium (Scigen Scientific, Gardena, CA). Embedded SC pieces were sectioned into 14 μm thick coronal slices using a Leica cryostat (Leica Biosystems, Nussloch, Germany). Antigen retrieval was performed using a citrated buffer (10 mM Citric acid containing 0.05% Tween-20, pH6.0). Sections were stained as recommended by the antibody manufacturer. Nonspecific Ab binding was blocked using 5% bovine serum albumin and 10% serum corresponding to the host of the secondary antibody with 0.3% Triton X-100 for 2 h at room temperature. Primary antibodies were diluted in blocking buffer and incubated overnight at 4 °C. After thorough washing with PBS, sections were incubated with secondary antibodies for 1–2 h at room temperature. Primary antibodies included mouse anti-IBA1 (1:250, generated at the Cleveland Clinic Hybridoma Core, provided by Bruce Trapp), rabbit anti-ionized calcium binding adaptor molecule 1 (IBA1) (1:250, 019-19741; Wako, Richmond, VA), rat anti-mouse CD3 (1:100, eBioscience, 16-0032-85,), rabbit anti-amyloid precursor protein (APP) (1:100, ThermoFisher Scientific, 51-2700), rat anti-major histocompatibility complex II (MHCII) (1:200, Abcam, ab139365), rabbit anti-active caspase 3 (1:200, Abcam ab2302), rat anti-CD68 (1:200, BIO-RAD, MCA1957), rabbit anti-degenerated myelin basic protein (dMBP) (1:2000, Millipore Sigma, AB5864), rabbit anti-Ki67 (1:200, Abcam, ab15580). Secondary antibodies (1:1000) were Alexa Fluor 488 goat anti-rat IgG (Invitrogen, A11006), Alexa Fluor 488 donkey anti-rat IgG (Invitrogen, A21208), Alex Flour 488 goat anti-mouse IgG (Invitrogen, A11006), Alexa Fluor 555 goat anti-rabbit IgG (Invitrogen, A21428), Alexa Flour 555 donkey anti-rabbit IgG (Invitrogen, A31573), and Alexa Flour 647 donkey anti-mouse IgG (Invitrogen, A31571). Demyelination was determined using Fluoromyelin™ Red Fluorescent myelin stain (FM, 1:300, Invitrogen, F34652) after secondary antibody staining. After washing, sections were mounted with ProLong™ Gold antifade Mountant with DAPI (Invitrogen, P36935). Images were acquired using the 20× objective of a Zeiss LSM 800 confocal laser scanning microscope equipped with Zen 2.3 software (Carl Zeiss, Jena, Germany). Images shown are representative of four to six sections from cervical to lumbar regions of SCs from 3 mice per timepoint and group. All images were taken from demyelinated lesions in the ventral funiculus of SC and were analyzed using Fiji version 1.0 (NIH). Demyelinated lesions in SC were measured from the white matter area showing loss of FM. The numbers of CD3-, Ki67- and active caspase3-immunopositve cells, and pyknotic cells were counted in 319.45 μm^2^ regions of the demyelinated ventral funiculus and the average numbers were used to obtain a count of cells per mm^2^ for each lesion. Pyknotic nuclei were identified based on DAPI staining pattern which showed condensed chromatin. Volume of dMBP-, CD68-, APP- and MHCII-positive cells were measured in 319.45 μm × 319.45 μm × 14 μm to acquire the volume of positive cells per mm^3^ using the voxer measuring program in Fiji version 1.0 (NIH). A total number of 12–18 lesions from 3 mice (4–6 lesions/mouse) were analyzed, with each lesion represented by a circle in the respective graphs.

### Statistical analysis

Statistics were determined using unpaired two-tailed Student *t* test and verified using two-way analysis of variance (ANOVA) with Bonferroni post-test. *P* values of clinical scores were determined by Mann–Whitney unpaired *t* test. Graphs were plotted using a Graphpad Prism 8.4 software (Graphpad Software, Inc., LA Jolla, CA).

## Results

### *Trem2* expression is prominently increased in SC infiltrating BMDM during MHV–JHM CNS infection

**Fig. 1:**
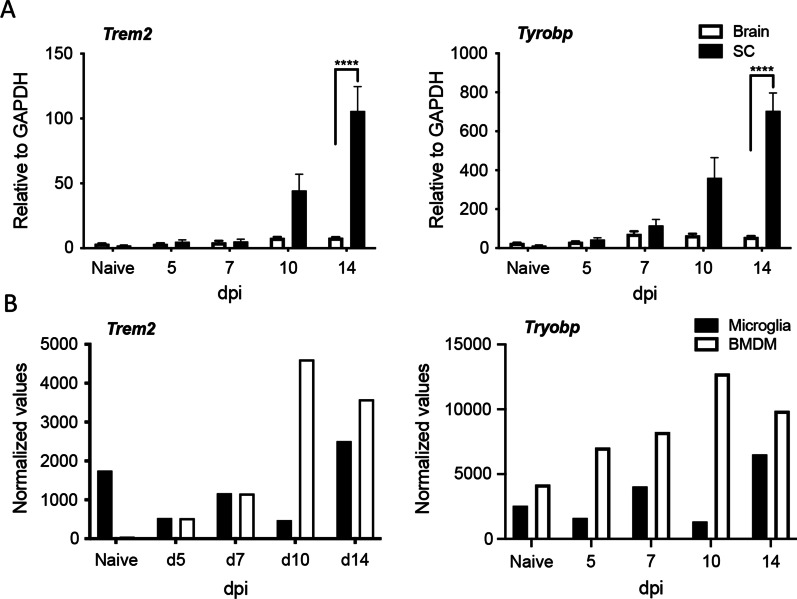
*Trem2* expression is prominently increased in SC infiltrating BMDM during MHV–JHM CNS infection. **A** *Trem2* and *Tyrobp* mRNA expression in brains and SCs from 5–6 mice at days 0, 5, 7, 10 and 14 pi after intracranial MHV–JHM infection. Data show the mean ± SEM from 2 independent experiments (*n* = 3–4 mice/timepoint/experiment) and are analyzed by unpaired two-tailed Student *t* test and two-way ANOVA analysis followed by Bonferroni test. ****p < 0.00001. **B** Normalized *Trem2* and *Tyrobp* mRNA expression from Nanostring nCounter analysis of microglia and BMDM sorted from pooled SC of 5–7 mice at days 0, 5, 7, 10 and 14 pi from our previously published data (GSE214472) [22]

**Fig. 2:**
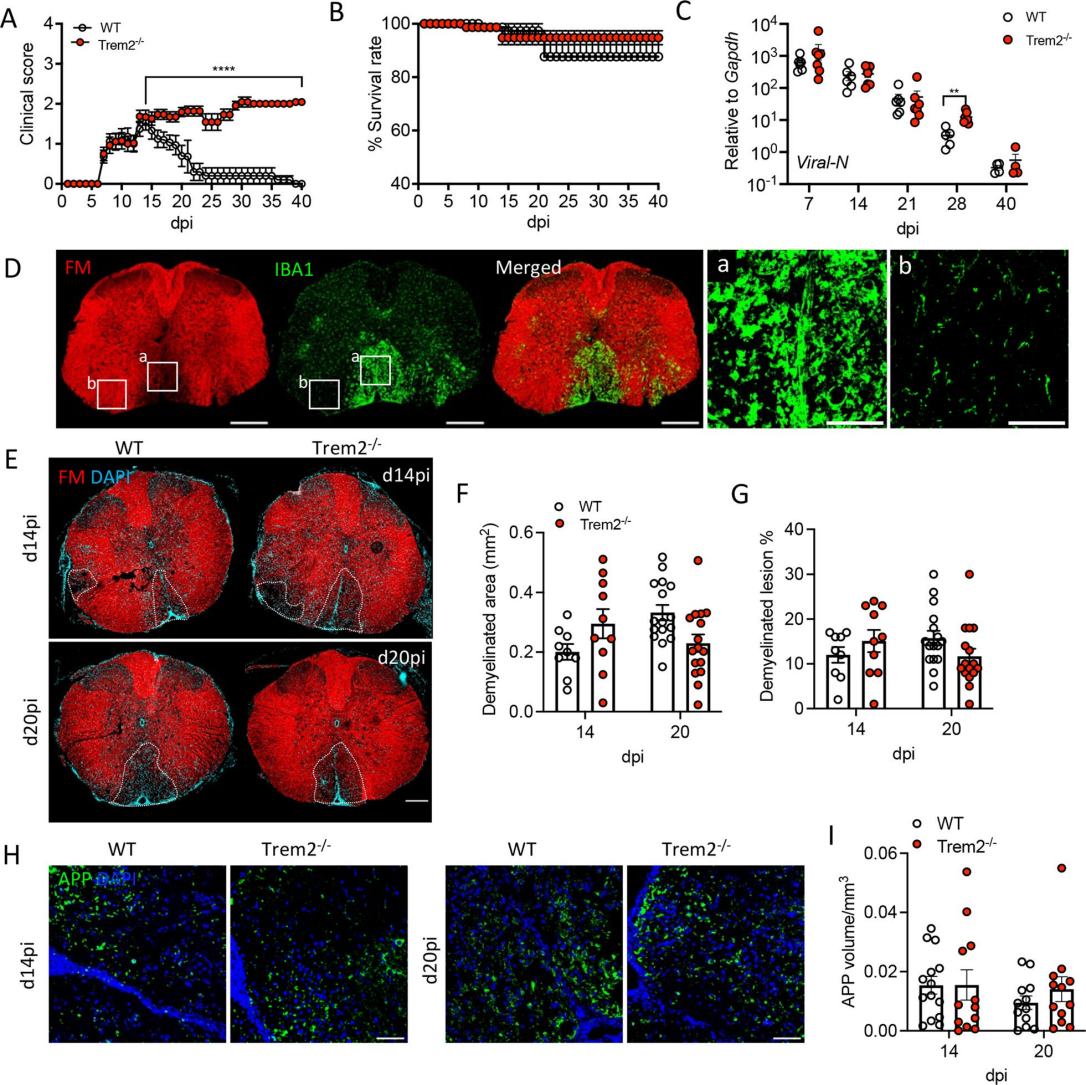
Failed recovery of diseased *Trem2*^*−/−*^-infected mice is not associated with worsened demyelination or axonal damage. Disease progression (**A**) and mortality (**B**) of MHV–JHM-infected WT (*n* = 24) and *Trem2*^−/−^ (*n* = 30) mice. *p* values of clinical scores were determined by Mann–Whitney unpaired *t* test. **C** Time course of virus replication monitored by *Viral-N* mRNA levels using RT-PCR. Data are the mean ± SEM from 2 independent experiments (*n* = 3–4 mice/timepoint/experiment) and are analyzed by unpaired two-tailed Student *t* test and two-way ANOVA analysis followed by Bonferroni test. **p < 0.001, ****p < 0.00001. **D** Representative images of a demyelinated lesion in a coronal WT mouse SC section detected by loss of FM staining (red) and accumulation of active IBA1 positive microglia/BMDM (green) at day 20 pi. Scale bar = 200 μm. Zoomed-in images show a demyelinated (a) and non-lesioned area (b). Scale bar = 50 μm. **E** Representative images showing staining of thoracic SC sections of MHV–JHM-infected WT and *Trem2*^−/−^ mice with FM (red) and DAPI (cyan) at days 14 and 20 pi. White-dotted lines outline the demyelinated area. Scale bar = 100 μm. **F** Lesion size per mm^2^ and **G** proportion of SC white matter demyelination. **H** Representative images of axonal damage by staining with anti-APP Ab (APP:red, DAPI:blue); **I** Volume of APP positive area per μm^3^ in the lesions. Scale bar = 50 μm. Data show the mean ± SEM from 12 to 18 lesions from 3 mice (4–6 lesions/mouse) per timepoint and are analyzed by unpaired two-tailed Student *t* test and two-way ANOVA analysis followed by Bonferroni test. Individual lesions are represented by a circle

Trem2 expression is a hallmark of microglia in the murine CNS known to limit neurodegenerative diseases by uptake and degradation of amyloid deposition as well as damaged myelin [1]. Although *Trem2* mRNA is highly expressed in microglia compared to circulating monocytes in naïve mice [22], BMDM upregulate *Trem2* in response to tissue damage [22]. BMDM, which are rapidly recruited to the CNS during MHV–JHM infection may thus also contribute to Trem2 functions, specifically phagocytosis of damaged myelin [22, 34]. We, therefore, assessed how *Trem2* and *Tyrobp* mRNA levels in brains and SCs are altered by infection, and correlate with BMDM accumulation. *Tyrobp* mRNA encodes TYROBP/DAP12, the TREM2 adaptor protein DNAX activation protein 12. Both *Trem2* and *Tyrobp* mRNA were low but detectable in the brain and SC in naïve animals (Fig. 1A). Upon MHV–JHM infection, *Trem2* and *Tyrobp* mRNA levels were not altered at days 5 and 7 post infection (pi) (Fig. 1A), although BMDM constitute the most prominent brain infiltrating leukocyte population at this timepoint [19]. *Trem2* and *Tyrobp* mRNA levels further remained unaltered in brains at days 10 and 14 pi, but their expression significantly increased in SCs at day 10 p.i., and even more prominently at day 14 pi (Fig. 1A). Analysis of our published SC derived microglia and BMDM nCounter Nanostring mRNA expression data throughout MHV–JHM infection [22] revealed that *Trem2* mRNA levels in microglia were not substantially altered during acute infection, but slightly increased at day 14 pi coincident with demyelination (Fig. 1B). In contrast, SC-infiltrated BMDM increased *Trem2* transcripts by 22-fold at day 7 pi and greater than 70-fold by days 10 and 14 pi, compared to circulating monocytes in naïve mice (Fig. 1C). Basal expression levels of *Tyrobp* mRNA were higher in naïve BMDM than microglia. Infection elevated *Tyrobp* transcripts throughout days 5 to 10 pi in BMDM, but not in microglia; both cell types had elevated transcript levels at day 14 pi (Fig. 1B) coincident with demyelination [35]. Overall, these data support a correlation between *Trem2* upregulation, which was more robust in SC-infiltrating BMDM than microglia, and MHV–JHM-induced demyelination evident by day 14 pi (Fig. 2D–F). However, it remains unclear whether microglia and BMDM exert similar or distinct functions depending on DAP12 signaling or release of sTrem2. sTrem2 may not only reduce membrane Trem2 activity, but also cause direct effects as a decoy receptor or by binding to ligands on other cells [12, 36, 37].

### *Trem2*^−/−^ mice fail to recover from MHV–JHM-induced clinical disease but do not exhibit worsened demyelination or axonal damage

Trem2 has numerous context-dependent immune regulatory functions apart from promoting myelin clearance, including limiting proinflammatory mediators. We thus determined the effects of Trem2 deficiency on MHV–JHM control and pathogenesis. Disease onset and timing to peak clinical disease (around day 14 pi) were comparable between WT and *Trem2*^*−/−*^ mice (Fig. 2A). However, whereas WT mice gradually recovered, *Trem2*^*−/−*^ mice exhibited sustained disease severity over day 40 pi marked by wasting and inability to upright position themselves (Fig. 2A). Nevertheless, Trem2 deficiency did not affect the survival rate (Fig. 2B). To assess whether sustained disease resulted from impaired virus control or sustained tissue damage, viral load was measured by viral nucleocapsid (*Viral-N*) mRNA levels. Similar overall *Viral-N* mRNA levels and rate of decline throughout days 7 to 21 pi indicated no defects in the control of acute viral replication. Although *Viral-N* mRNA decreased further by day 28 pi in WT mice, it remained slightly elevated in *Trem2*^−/−^ mice, but reached similar levels in both groups by day 40 p.i. (Fig. 2C). Importantly, we could not detect infectious viral titers by plaque assay at day 21 pi in either group (data not shown), suggesting infectious virus remained below detection limits at day 28 pi. The inability of *Trem2*^*−/−*^ mice to recover, already apparent after day 14 pi in WT mice, could thus not be attributed to compromised virus control.

MHV–JHM-induced SC lesions detected by loss of FM are mainly found in the ventral funiculus, to some extent in lateral white matter tracks, and occasionally in the dorsal funiculus (Fig. 2D). IBA1 staining to gauge microglia/macrophage activation showed highly increased immunoreactivity in demyelinated lesions (Fig. 2Da) contrasting low immunoreactivity in non-lesioned white matter (Fig. 2Db). The high density of IBA1^+^ cells in lesions preempted differentiation between microglia and BMDM based on morphology (Fig. 2D). Staining with the anti-transmembrane 119 (Tmem119) Ab to specifically identify microglia [38] only revealed rare Tmem119^+^IBA^+^ cells in lesions (Additional file 1: Fig. S1), consistent with diminished Tmem119 expression in demyelinating lesions noted in other studies [39–41]. Overall, the activated IBA1^+^ cells in lesions support that the vast increase of *Trem2* transcripts in infected SCs is attributed to lesion-localized BMDM and microglia.

The loss of Trem2 worsened demyelination and axonal damage in both the CPZ-induced as well as EAE demyelination models [7, 8, 10]. Therefore, we determined whether Trem2 deficiency affects virus-induced demyelination. *Trem2*^*−/−*^ mice showed no overt changes in the overall distribution of lesions, the percent of demyelinated white matter or lesion size compared to WT mice (Fig. 2E–G). To assess if impaired disease recovery of *Trem2*^*−/−*^ mice was attributed to enhanced axonal damage, we determined the accumulation of APP, a marker reflecting impaired axonal transport in injured axons [42]. APP-positive areas were restricted to demyelinated lesions in both WT and *Trem2*^*−/−*^ mice, albeit with no difference in the staining pattern or abundance between the groups at either day 14 or 20 pi (Fig. 2H, I). These data suggested that Trem2 deficiency associated with prolonged clinical disease in the time interval analyzed is not associated with overt worsening of MHV–JHM-induced demyelination or axonal damage, distinct from the CPZ-induced or EAE demyelination models [7, 10, 11].

### Trem2 ablation results in less proliferation and more apoptotic cells in demyelinated lesions

**Fig. 3:**
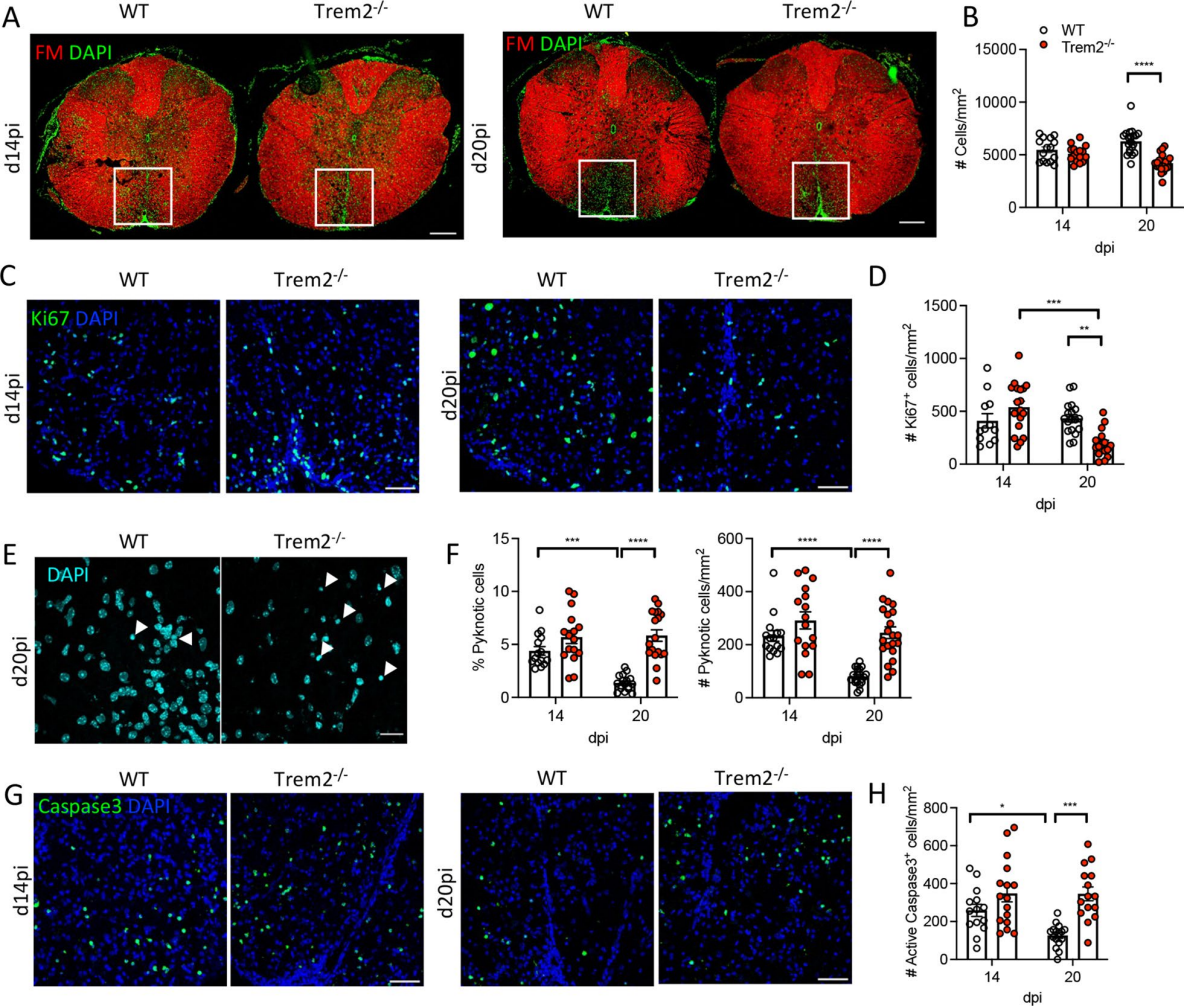
Trem2 ablation results in reduced cellular proliferation and increased apoptosis within demyelinated lesions. **A** Representative images of cellularity in SC demyelinated lesions from WT and *Trem2*^−/−^ shown by FM (red) and DAPI (green) staining at days 14 and 20 pi. Scale bar = 200 μm. **B** Counts of total DAPI positive cell numbers measured in the white box (319.45 μm × 319.45 μm) of (**A**). **C** Representative images of anti-Ki67 mAb (green) and DAPI (blue) staining. Scale bar = 50 μm. **D** Counts of Ki67^+^ cells per mm^2^. **E**, **F** Representative images, number and proportion of pyknotic nuclei per mm^2^ demyelinated lesion area from WT and *Trem2*^−/−^ mice. Arrow points depict pyknotic nuclei. **G** Representative images of active-caspase 3 (green) and DAPI (blue) staining. Scale bar = 50 μm. **H** Counts of active-caspase 3^+^ cells per mm^2^. Data show the mean ± SEM from 12 to 18 lesions from 3 mice (4–6 lesions/mouse) per timepoints and are analyzed by unpaired two-tailed Student *t* test and two-way ANOVA analysis followed by Bonferroni test. *p < 0.01, **p < 0.001

Trem2 and DAP12 promote cell proliferation and survival by attenuating apoptotic signals [4, 12]. This is supported by inhibited microglia proliferation in the corpus callosum during CPZ-induced demyelination in *Trem2*^*−/−*^ mice [7]. In MHV–JHM-induced demyelination, DAPI staining also revealed reduced cellularity in virus-induced demyelinated SC lesions of *Trem2*^−/−^ relative to WT mice at day 20 pi (Fig. 3A, B). We, therefore, determined how Trem2 influences proliferation and apoptosis of cells in lesions. At day 14 pi both WT and *Trem2*^−/−^ mice revealed comparable proliferating cells marked by Ki67 expression (Fig. 3C, D). However, while WT mice showed sustained cell proliferation at day 20 pi, *Trem2*^−/−^ mice exhibited a significant decline of Ki67 positive cells (Fig. 3C, D). To assess whether cell apoptosis is additionally affected by Trem2 deficiency, we monitored pyknotic nuclei as well as anti-active-caspase3 antibody reactivity. Pyknotic nuclei, characterized by condensed chromatin, are an indicator of dying cells in demyelinated lesions following MHV–JHM infection [24]. Consistent with this finding, we observed pyknotic nuclei in lesions of both WT and *Trem2*^*−/−*^ mice at day 14 pi with no apparent differences in numbers (Fig. 3F). However, while the numbers and proportion of pyknotic nuclei in WT mice were significantly reduced by day 20 pi, their numbers were sustained between days 14 and 20 pi in *Trem2*^*−/−*^ mice (Fig. 3E, F). In addition, both WT and *Trem2*^−/−^ mice also revealed comparable active-caspase3 positive cells at day 14 pi (Fig. 3G, H). However, WT mice showed diminished apoptosis at day 20 pi, whereas *Trem2*^−/−^ mice exhibited sustained higher numbers of active-caspase3 positive cells (Fig. 3G, H). Naïve uninfected WT mice showed very few proliferating or apoptotic cells in the ventral funiculus white matter areas of SC (Additional file 2: Fig. S2). Overall, these data indicate that the reduced cellularity in lesions of *Trem2*^*−/−*^ mice can be attributed to both limited proliferation and increased apoptosis during disease progression. Demyelinated lesions harbor diverse cell types, including microglia/BMDM, astrocytes, T cells, oligodendrocytes and their progenitors [35, 43–45]. Although apoptotic cells during MHV–JHM infection of WT mice are mainly lymphocytes [45], it remains to be identified whether microglia/BMDM or other cell types are indirectly affected by Trem2 ablation.

### Trem2 ablation does not alter SC immune cells recruitment despite mild alterations of pro- and anti-inflammatory factor expression

**Fig. 4:**
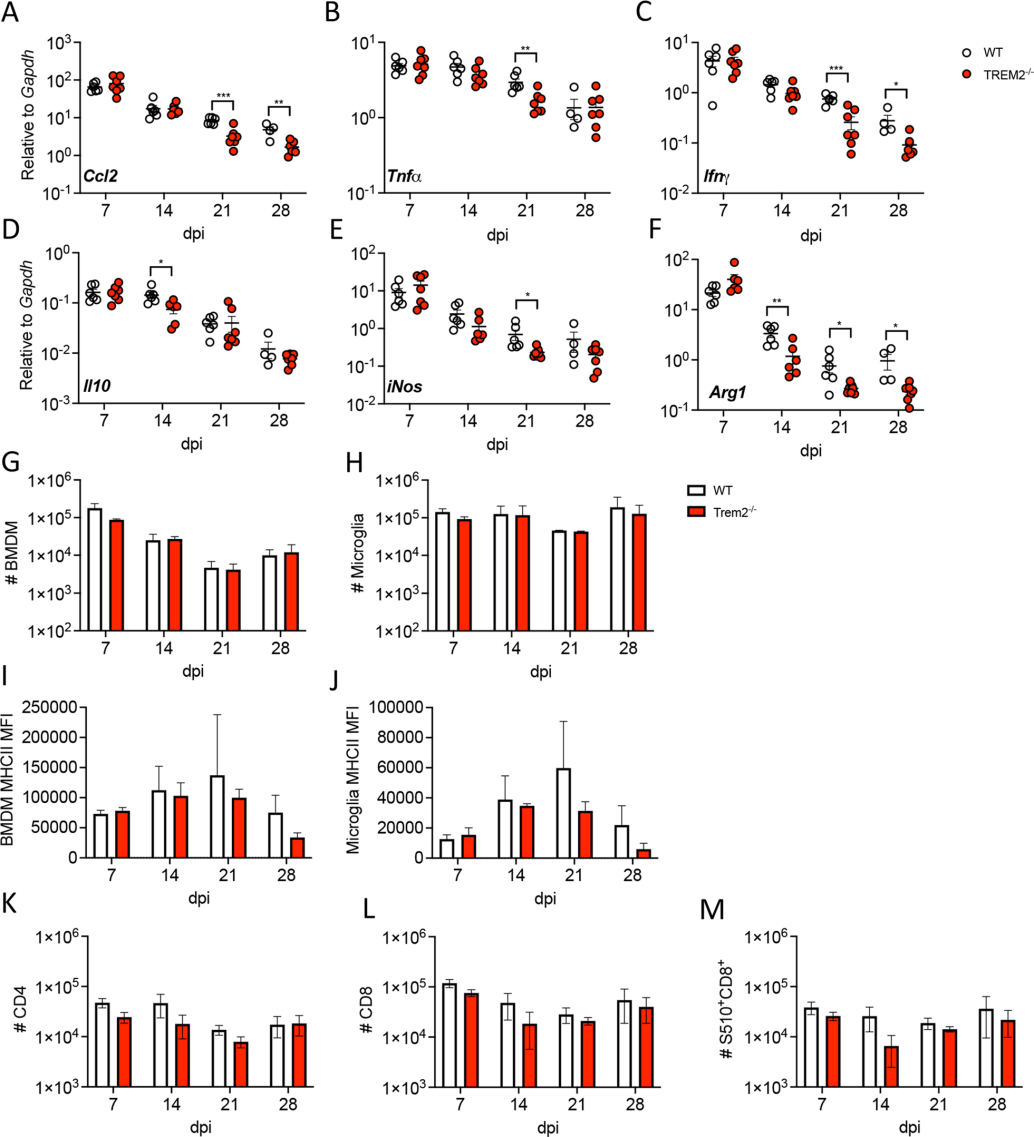
Trem2 ablation does not impair immune cell recruitment into the SC or myeloid cell activation. SC harvested from MHV–JHM-infected mice after PBS perfusion were used for RNA isolation and RT-PCR analysis. **A**–**F** *Ccl2, Tnfa, Ifng, Il10, inos* and *Arginase1* mRNA expression monitored at different timepoint. Data are the mean ± SEM from 2 independent experiments (*n* = 4–7 mice/timepoint/experiment) and are analyzed by unpaired two-tailed Student *t* test and two-way ANOVA analysis followed by Bonferroni test. *p < 0.01, **p < 0.001, ***p < 0.0001. **G**, **H** Numbers of BMDM (CD45^hi^CD11b^+^Ly6G^−^) and microglia (CD45^hi^CD11b^+^Ly6G^−^) from SC of MHV–JHM-infected WT and *Trem2*^−/−^ mice were analyzed by flow cytometry at the indicated timepoints. **I**, **J** Surface expression of MHCII as assessed by mean fluorescent intensity (MFI) on BMDM or microglia. **K**–**M** Numbers of CD4, CD8 and virus-specific S510^+^ tetramer^+^ CD8 T cells from SCs of WT and *Trem2*^−/−^ mice. Data for flow cytometry are representative of three independent experiments, each with *n* = 3–4 individuals per timepoint and are analyzed by unpaired two-tailed Student *t* test and two-way ANOVA analysis followed by Bonferroni test

Trem2 has been shown to restrain macrophage/microglia activation by reducing proinflammatory mediators [3], but also to enhance activation of microglia in CPZ-induced demyelination [7, 9], indicating context dependent regulation. We, therefore, quantified SC mRNA levels encoding various pro-inflammatory and disease resolving factors, namely, the monocyte chemoattractant CCL2, TNF, IFNγ, a prominent mediator of MHV virus control, iNOS, IL10 and arginase 1 (Arg1) over the course of infection. *Ccl2, Tnf, Ifnγ, inos, Il10* and *Arg1* mRNA levels were all similar in both groups during acute infection at day 7 pi (Fig. 4A–F). At day 14 pi, only *Il10* and *Arg1* mRNA levels, both considered disease resolving mediators, were reduced in the absence of Trem2, while mRNAs linked to proinflammatory factors, *Ccl2, Tnf, Ifnγ* and *inos* were similar in both *Trem2*^*−/−*^ and WT mice (Fig. 4A–F). Disease progression in *Trem2*^*−/−*^ mice at day 21 pi was associated with significantly reduced *Ccl2, Tnf, Ifnγ, inos* and *Arg1* mRNA; lower levels persisted out to day 28 pi for *Ccl2, Ifnγ* and *Arg1* mRNA. Overall the day 21 pi timepoint was most notably marked by reduced, rather than increased levels of pro-inflammatory factors in the absence of Trem2 reminiscent of the CPZ studies [7, 9].

**Fig. 5:**
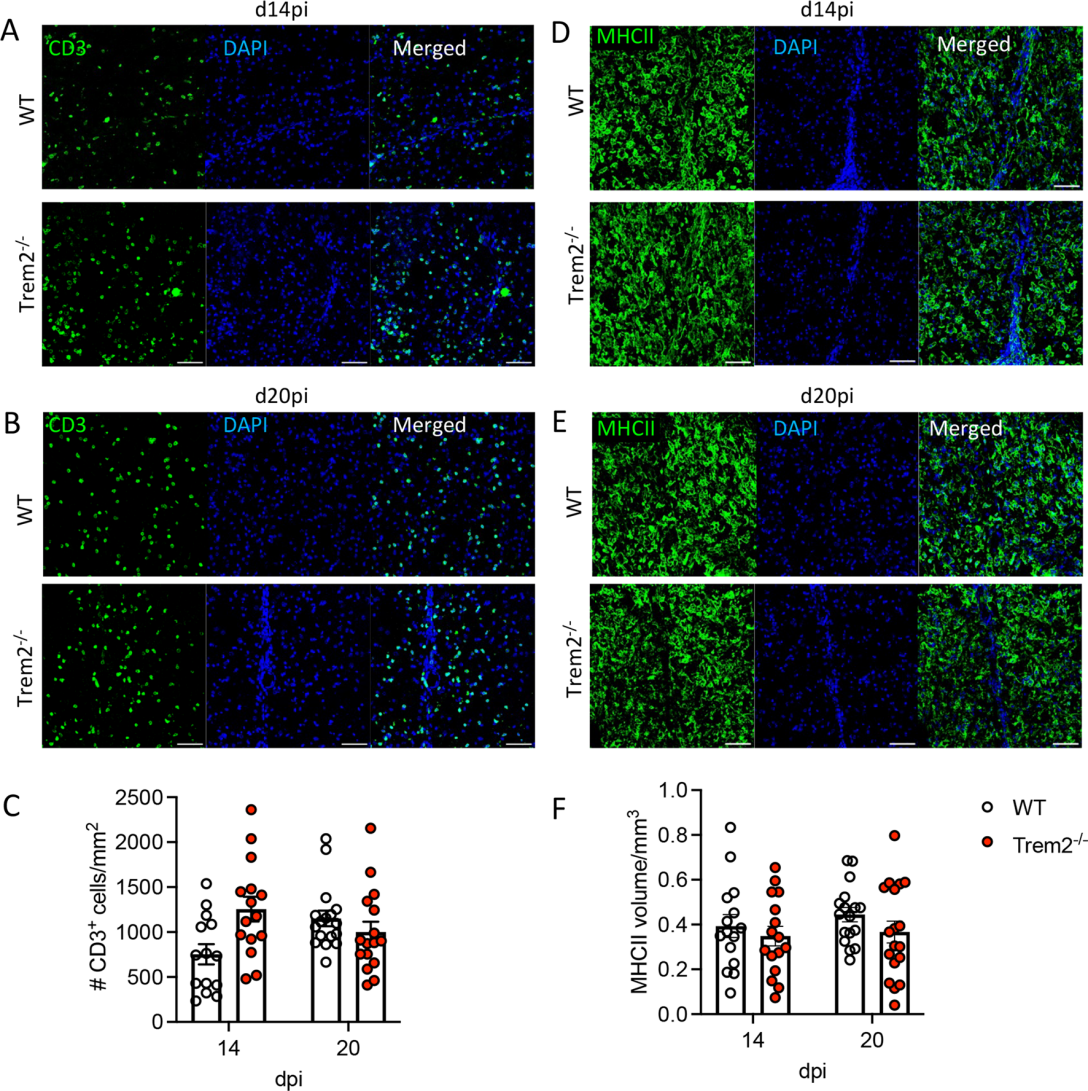
Trem2 ablation does not impair CD3 T cell infiltration or MHC class II expression in lesions. SC sections from MHV–JHM-infected WT and *Trem2*^*−/−*^ mice were analyzed for CD3^+^ lymphocyte distribution and MHC class II expression. **A**, **B** Representative images of CD3 (green) and DAPI (blue) positive cells in demyelinated lesions. **C** Quantification of CD3 numbers per mm^2^ of SCs form WT and *Trem2*^*−/−*^ mice at days 14 and 20 pi. **D**–**E** Representative images of MHCII (green) and DAPI (blue) staining in demyelinated lesions. Scale bar = 50 μm. **F** Quantification of the volume of MHCII positive cells per μm^3^ at days 14 and 20 pi. Data show the mean ± SEM from 12 to 18 lesions from 3 mice (4–6 lesions/mouse) per timepoint and are analyzed by unpaired two-tailed Student *t* test and two-way ANOVA analysis followed by Bonferroni test

To assess whether subtle differences in mRNA levels of pro-inflammatory and disease resolving mediators were associated with alterations in leukocyte recruitment, SCs from WT and *Trem2*^−/−^ mice were analyzed for leukocyte subsets by flow cytometry. No overt differences in BMDM recruitment were noted at any timepoint, despite lower *Ccl2* mRNA expression in the absence of Trem2 at later stages of disease; microglia numbers were also similar between groups (Fig. 4G, H). Furthermore, MHC class II surface expression as a maker for IFNγ signaling on BMDM/microglia was also not altered between the groups (Fig. 4I, J). Furthermore, we did not detect significant differences in numbers of CD4, CD8 or virus-specific S510^+^/D^b^ tetramer^+^ CD8 T cells throughout infection (Fig. 4K–M). CD4 and CD8 T cell effector functions are the major drivers of immune mediated demyelination following MHV infection [19]. To determine if sustained clinical symptoms are associated with anatomically localized differences, not evident by global flow cytometric analysis, SC sections were assessed histologically for local accumulation of T cells or MHC class II expression in lesions. CD3 positive cells were distributed throughout the parenchyma of demyelinated areas and no significant differences were noted between the groups at days 14 or 20 pi (Fig. 5A–C). We also assessed MHC class II expression in lesions by volumetric analysis as the ramified nature of microglia with thickened processes makes quantification of class II in one plane unreliable. The MHC class II positive volume was also similar at both days 14 and 20 pi (Fig. 5D–F), consistent with flow cytometry data. These data support that prolonged disease in *Trem2*^*−/−*^ mice is not attributed to altered T cell effector function/distribution, BMDM recruitment or activation of microglia/BMDM.

### Trem2 ablation impairs phagocytic activity of myeloid cells

**Fig. 6:**
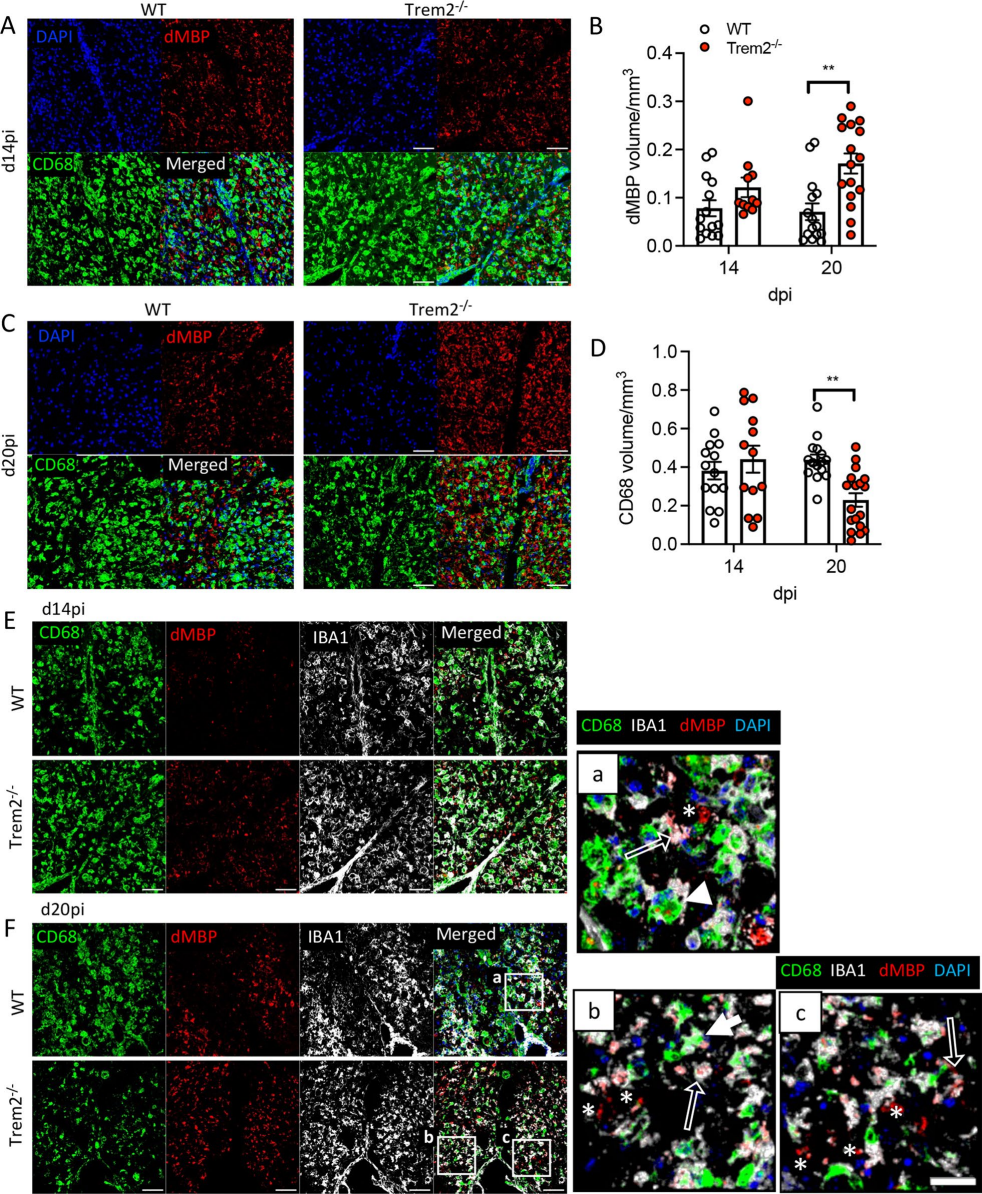
Trem2 ablation impairs uptake and degradation of myelin debris following virus-induced demyelination. **A**,** B** Representative images of dMBP (red), CD68 (green) and DAPI(blue) staining in SC demyelinated areas of WT and *Trem2*^*−/−*^ mice at days 14 and 20 pi. Scale bar = 50 μm. **C**, **D** Quantification of volume of dMBP positive area (**C**) and CD68 positive cells (**D**) per mm^3^. **E**, **F** Representative images of co-staining for dMBP (red), IBA1 (white) and CD68 (green) in lesions of WT and *Trem2*^*−/−*^ mice at days 14 and 20 pi. Scale bar = 50 μm. a–c Zoomed-in images of boxed areas in (**F**). Representative images of ‘free’ dMBP (asterisk), dMBP associated with only IBA1 (open arrow) and dMBP associated with both IBA1 and CD68 (white arrowhead). Scale bar = 20 μm. Data show the mean ± SEM from 12 to 18 lesions from 3 mice (4–6 lesions/mouse) per timepoint and are analyzed by unpaired two-tailed Student *t* test and two-way ANOVA analysis followed by Bonferroni test. **p < 0.001

Numerous studies show that Trem2 is involved in the uptake and degradation of myelin debris, a process essential for remyelination [7, 8, 10]. MHV–JHM infection mounts a highly polarized Th1 response to control virus infection, providing a very distinct local immune environment than the CPZ or EAE-induced demyelinating models. We, therefore, determined whether Trem2 is equally essential in clearing myelin debris using anti-damaged myelin Ab (dMBP) in combination with anti-IBA1 and -CD68 Abs to assess phagocytic activity. CD68 is a commonly used phagocytic marker of monocyte lineage cells, including tissue macrophages and microglia, which is expressed on late endosomes and lysosomes [6, 46]. Anti-dMBP Ab reactivity was detected exclusively in demyelinated lesions marked by loss of FM staining in both mouse groups (data not shown). Accumulation of dMBP was initially comparable in both groups; however, Trem2 deficiency resulted in significantly increased dMBP staining at day 20 pi relative to WT mice (Fig. 6A–C). CD68 staining showed a large and dense staining pattern (Fig. 6A, C, E, F) consistent with CD68 in phagocytic vesicles in foamy macrophages in MS lesions [47]. While the extent of CD68 expression was similar in both mouse groups at day 14 pi, there was reduced expression in *Trem2*^*−/−*^ mice by day 20 pi relative to WT mice, which sustained CD68 expression (Fig. 6A, C, D). Furthermore, we detected three different staining patterns for IBA1, dMBP and CD68 in demyelinated lesions from both WT and *Trem2*^−/−^ mice: ‘free’ dMBP (dMBP^+^CD68^−^IBA1^−^), dMBP engulfed by IBA1 cells without association with CD68 (dMBP^+^IBA1^+^CD68^−^) and dMBP engulfed by IBA1 cells and associated with CD68 (dMBP^+^IBA1^+^CD68^+^) in both mice (Fig. 6E, F, a–c). As astrocytes also have the capacity to uptake myelin debris [48, 49], we assessed this possibility using co-staining for dMBP and GFAP in *Trem2*^−/−^ mice. However, despite the presence of activated astrocytes in lesions, there was no evidence of substantial dMBP uptake by astrocytes (Additional file 3: Fig. S3). Increased ‘free’ dMBP in *Trem2*^*−/−*^ mice at day 20 pi (Fig. 6F, c) thus indicated inefficient uptake and clearance of damaged myelin in the absence of Trem2. These data are consistent with other studies demonstrating reduced uptake of amyloid beta (Aβ) by CD68 positive phagosomes in the Trem2 deficient AD mouse model [50]. This also implicates impaired capacity of phagolysosome formation and debris degradation. The apparent disparity between similar overall demyelination despite increased detection of dMBP in *Trem2*^*−/−*^ mice may be skewed by low magnification images showing loss of FM staining in entire SC sections, compared to high magnification images revealing punctate dMBP reactivity.

### Trem2 ablation is associated with impaired induction of select phagocytotic markers

**Fig. 7:**
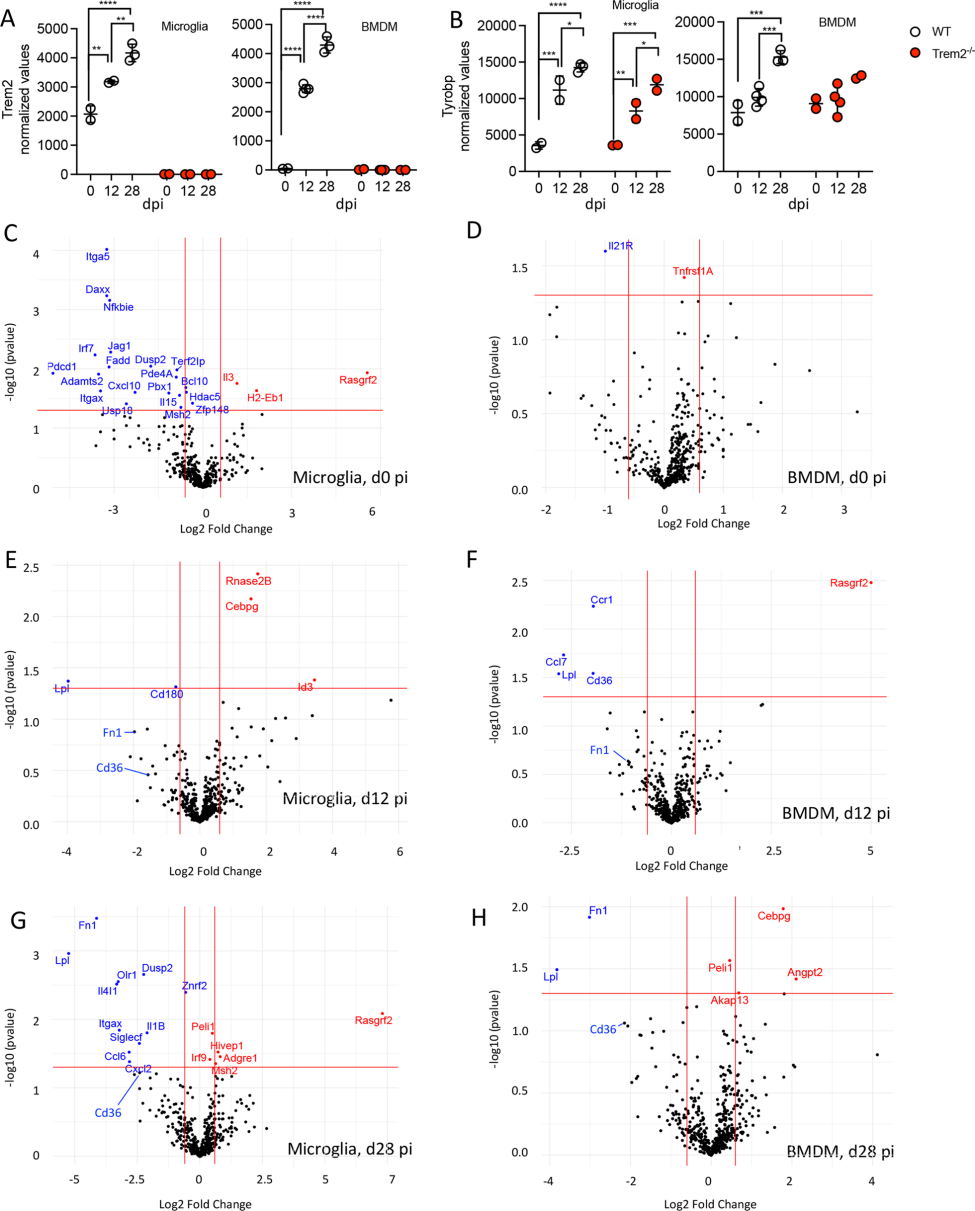
Differentially expressed genes in microglia and BMDM from *Trem2*^−/−^ mice compared to WT mice. Microglia (CD45^int^CD11b^+^) and BMDM (CD45^hi^CD11b^+^Ly6G^−^) were purified from blood (day 0 pi), and SCs of infected WT and *Trem2*^−/−^ mice at days 12 and 28 pi by FACS; RNA was subjected to nCounter analysis using the myeloid cell probe panel. **A**, **B** Normalized count values of *Trem2* and *Tyrobp* mRNA expression in microglia and BMDM from both mouse groups at indicated timepoints. Individual dots represent cells from an individual mouse for BMDM at day 12 pi and cells pooled from 2 to 3 mice at days 0 and 28 pi for both microglia and BMDM. Data show the mean ± SEM from 4 individual or 2–3 pooled samples per timepoint and are analyzed by unpaired two-tailed Student *t* test and two-way ANOVA analysis. **C**–**H** Volcano plots depict differentially expressed genes (*p* < 0.05) in microglia (**C**, **E**, **G**) and BMDM (**D**, **F**, **H**) from *Trem2*^−/−^ relative to WT mice at days 0, 12 and 28 pi. *Trem2* was excluded from the volcano plots. Horizontal and vertical red lines guide *p* value < 0.05 and log2FC >|0.5, respectively

To assess potential dysregulation of other genes associated with phagocytosis and degradation or proinflammatory mediators in the absence of Trem2, we compared gene expression profiles SC-derived microglia and BMDM from WT and *Trem2*^−/−^ mice isolated by flow cytometry (Additional file 4: Fig. S4). We focused on two timepoints, namely, the peak of disease (day 12 pi) coincident with demyelination and similar clinical scores between groups and a late timepoint (day 28 pi). Day 28 pi was chosen over day 20 pi to capture differences in gene expression when clinical scores were most divergent, i.e., WT mice had fully recovered, but *Trem2*^*−/−*^ mice showed disease progression. Microglia and blood-derived monocytes from naïve animals were used to establish relative baseline values. mRNA profiles from FACS enriched populations were analyzed using the Nanostring nCounter platform and the myeloid gene panel. Initial analysis of *Trem2* and *Tryobp* transcripts confirmed the absence of *Trem2* transcripts in *Trem2*^*−/−*^ mice (Fig. 7A) and revealed dramatic upregulation of *Trem2* and *Tryobp* transcripts in both microglia and BMDM from WT mice during demyelination (Fig. 7A, B). *Tyrobp* mRNA was also highly upregulated in microglia and to a lesser extent in BMDM from WT mice. Interestingly, Trem2 deletion still resulted in upregulation of *Tyrobp* transcripts in microglia, but not in BMDM, suggesting differential regulation of *Tryobp* in microglia and BMDM [51, 52].

Volcano plots show overall differentially expressed genes (DEGs) in myeloid populations from *Trem2*^*−/−*^ mice relative to WT mice across the different timepoints (Fig. 7C–H). In naïve mice, Trem2 deletion was associated with downregulation of 21 genes including *Itgax* in microglia, but overall gene expression patterns in BMDM from *Trem2*^−/−^ and WT mice were similar; only very few genes, including *Tnfrsf11a* and *Il21r* out of 764 genes in the myeloid panel were affected by Trem2 deletion (Fig. 7C, D). During the peak of disease both microglia and SC-derived BMDM from *Trem2*^−/−^ mice showed similar numbers of significantly DEGs (Fig. 7E, F). More dynamic changes were evident at day 28 pi, with increased numbers of DEGs in microglia than BMDM, implicating different roles of Trem2 in microglia versus BMDM during the recovery/progressive disease phase (Fig. 7G, H). The two most significantly downregulated genes in both *Trem2*^*−/−*^ microglia and BMDM at day 28 pi were lipoprotein lipase (*Lpl*) and fibronectin 1 (*Fn1*) (Fig. 7G, H). Both microglia and BMDM from *Trem2*^*−/−*^ mice further expressed lower levels of gene which encodes the scavenger receptor CD36, also known as fatty acid translocase at day 28 pi (Fig. 7G, H). At day 12 pi, significantly lower levels of *Cd36* mRNA were specifically noted in *Trem2*^*−/−*^ BMDM (Fig. 7F). Expression of *Olr1*, *Ccl6* and *Itgax* genes was specifically downregulated at day 28 pi in *Trem2*^*−/−*^ microglia but not BMDM (Fig. 7G, H). LPL, FN1 and CD36 are all associated with lipid metabolism and phagocytic activities under neurogenerative conditions [6, 53, 54]; the *Olr1* gene encodes lectin-like oxidized low-density lipoprotein 1 (LOX-1) which internalizes and degrades oxidized low density lipoprotein [55, 56], whereas CCL6 is a chemotactic for monocytes, macrophages and T cells, that also improves macrophage phagocytic functions [57]. ITGAX, a receptor for fibrinogen, also plays a role in cellular adhesion, phagocytosis and chemotaxis [58].

**Fig. 8:**
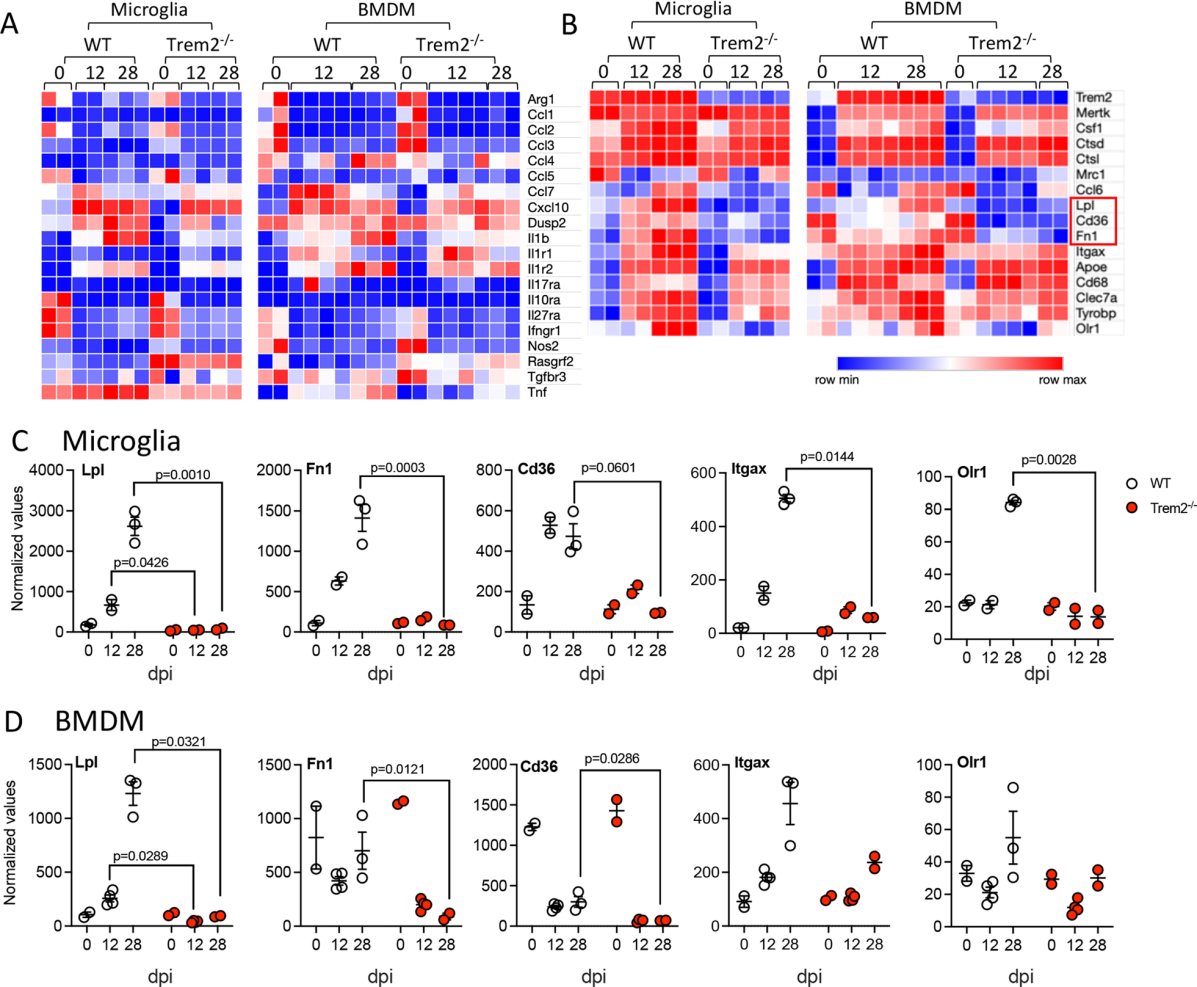
Trem2 ablation dysregulates expression of phagosome associated genes during MHV–JHM-induced demyelination**.** RNA from microglia (CD45^int^CD11b^+^) and BMDM (CD45^hi^CD11b^+^Ly6G^−^) from SCs of WT and *Trem2*^−/−^ mice at days 12 and 28 pi was subjected to nCounter analysis as depicted in Fig. 7. **A**, **B** Heatmaps show comparative expression of genes associated with inflammatory factors (**A**) and phagocytosis (**B**) in microglia and BMDM from WT and *Trem2*^−/−^ mice over time. **C**, **D** Normalized count value of *Lpl*, *Cd36*, *Fn1*, *Itgax* and *Orl1* mRNA expression in microglia and BMDM from WT and *Trem2*^−/−^ mice over timepoint. Individual dots indicate cell populations from individual mice (for BMDM at day 12 pi) or from 2 to 3 pooled mice (for microglia and BMDM at days 0 and 28 pi). Data for show the mean ± SEM of each cell population from 4 individual or 2–3 pooled mouse per timepoint and are analyzed by unpaired two-tailed Student *t* test and two-way ANOVA analysis

Heatmaps focusing on inflammatory molecules and their receptors did not reveal significant changes in most genes, with the exception of reduced *IL-1β* and *Tnf* in *Trem2*^*−/−*^ microglia and BMDM (Fig. 8A). As impaired dMBP clearance was the most prominent phenotype in infected *Trem2*^*−/−*^ mice and volcano plots revealed scavenger receptors and phagocytosis pathway genes as most significant DEG, we further generated heatmaps focusing on the phagocytosis pathway. Expression of *Lpl*, *Fn1* and *Cd36* were downregulated in both microglia and BMDM from *Trem2*^−/−^ mice relative to WT mice overtime after infection (Fig. 8B). Since volcano plots and heatmaps both show the relative expression between groups, we also plotted normalized gene counts to assess whether these genes were never induced or actively downregulated during infection relative to homeostatic levels. Trem2 ablation did not influence the basal levels of *Lpl, Fn1 or Cd36* transcripts in either microglia or BMDM (Fig. 8C, D). Interestingly, WT microglia dramatically increased *Lpl, Fn1* and *Cd36* expression after infection, whereas Trem2 ablation resulted in the complete failure to upregulate these genes in microglia (Fig. 8C). In addition, WT BMDM also upregulated *Lpl* transcripts following infection, but transcriptional activation was inhibited in the absence of Trem2. We previously showed that *Fn1* is expressed mainly in microglia but not in BMDM in MHV–JHM-infected SC [22]. Indeed, although *Fn1* and *Cd36* transcripts were higher in naïve BMDM relative to microglia in WT mice, expression levels of both genes were reduced in BMDM during chronic infection; the decrease in *Cd36* mRNA was notably more dramatic in BMDM from *Trem2*^−/−^ mice (Fig. 8D). *Itgax* expression was also significantly increased both microglia and BMDM from WT mice; however, its expression was not obvious in *Trem2*^−/−^ mice at day 28 pi (Fig. 8C, D). Upregulation of Olr1 expression was observed in foamy macrophages in active rims of demyelinating lesion of MS patient [55]. Trem2 ablation inhibits expression of Olr1 in microglia, but was only mildly affected in BMDM in MHV–JHM-infected mice (Fig. 8C, D). These results reveal similar regulation of *Lpl and Orl1*, yet opposing regulation of *Fn1*, *Cd36* in microglia relative to BMDM in WT mice.

## Discussion

Trem2 is an immune receptor with pleiotropic functions expressed prominently on microglia in the CNS [1, 4, 5], but also on tissue macrophages in various organs [12, 13, 15, 59, 60]. Trem2 acts as a sensor for various ligands including damage-associated lipids [61]. Activation and signaling through its adapter Tyrobp/Dap12 can regulate multiple functions including survival, phagocytosis, inflammatory responses, glycolysis and mTOR signaling [1, 3, 4, 51, 62]. While animal models of AD and MS support a disease attenuating role of Trem2 expressing microglia, studies examining a role of Trem2 during viral infections, which rely on proinflammatory factors for viral control, reveal context dependent protective as well as pathogenic functions [12, 13, 15]. Using neurotropic MHV–JHM infection as a model of acute encephalomyelitis resolving into a persistent infection associated with demyelination, our studies showed that Trem2 deficiency did not affect viral control or establishment of persistence. The failure to recover from clinical disease was rather associated with accumulation of damaged myelin similar to toxin and autoimmune-induced demyelinating models.

Distinct from no overt effect on MHV replication (Fig. 2), Trem2 directly facilitated virus replication of porcine reproductive and respiratory syndrome virus in macrophages via interaction with a viral nonstructural protein and suppressed protective proinflammatory responses [13]. However, no adverse effects of Trem2 deficiency on viral control during Sendai virus infection of the lung [12] or LCMV liver infection [15] is congruent with our data. Trem2 deficiency actually accelerated LCMV virus clearance, diminished apoptosis, and ameliorated liver damage, despite similar T cell responses [15]. T cell accumulation and anti-viral function in the MHV–JHM-infected CNS were also not affected by Trem2 deficiency (Fig. 4), suggesting that Trem2 does not overtly affect T cell activation in viral infections. This also appears to be the case in more limited inflammatory settings associated with AD. The cerebrospinal fluid from AD patients harbors clonally expanded CD8 T cells [63]; however, Trem2 deficiency in an AD mouse model did not alter CNS T cell numbers [64]. By contrast, a recent study demonstrated that Trem2 upregulation on CD4^+^ T cells promoted Th1–mediated host defense against Mycobacterium tuberculosis infection in both mice and humans [65]. Conditional depletion of Trem2 in CD4 T cells as well as transfer of Trem2 deficient CD4 T cells into Rag^−/−^ mice resulted in increased bacterial load and more severe lung pathology relative to Trem2 sufficient counterparts [65]. These results imply that the metabolite and proinflammatory milieu at the site of initial T cell activation can influence T cell activity via both Trem2 expressing myeloid cells or ligands engaging Trem2 on T cells.

The hallmark of MHV pathogenesis is demyelination as a result of T cell mediated anti-viral effector function [19]. Unlike WT mice, which rapidly recover coincident with removal of damaged myelin, the accumulation of damaged myelin in the absence of Trem2 correlated with worsening clinical disease (Fig. 2). Sustained clinical disease could not be attributed to dysregulation of acute immune responses, virus control, or altered viral RNA persistence (Figs. 2 and 4). Similarly, in Sendai virus infection, a role for Trem2 only became evident post virus control and Trem2 deficiency ameliorated chronic inflammatory respiratory disease by mitigating sTrem2 enhanced macrophage survival [12]. By contrast, increased apoptosis and pyknotic nuclei over time in the absence of Trem2 during persistent MHV infection (Fig. 3) suggests that worsened pathogenic outcome correlated most prominently with impaired removal of damaged myelin as well as apoptotic cells. Although Trem2 ablation did not affect lesion size, sustained accumulation of damaged myelin was consistent with findings in toxin and autoimmune-induced demyelination models [7, 8, 10]. Similar to our RNA profiling data, impaired microglial activation and myelin debris clearance in *Trem2*^−/−^ mice in the CPZ model were associated with compromised gene expression related to microglia activation, phagocytosis and lipid metabolism [7]. Trem2 agonist treatment in the CPZ model accelerated myelin uptake and degradation, thereby promoting oligodendrocyte precursor density, oligodendrocyte maturation, and remyelination resulting in preservation of axonal integrity [10]. In lysolecithin-induced demyelination, Trem2 deletion did not alter the amount of myelin debris in lysosomes, but resulted in decreased myelinated axons, oligodendrocytes, as well as foamy macrophages, therefore, impairing regenerative responses [66] similar to the CPZ model. Trem2 deficiency in an AD mouse model further showed reduced uptake of Aβ by CD68 positive phagosome [50]. Consistent with the latter study, defective clearance of damaged myelin was associated with decreased CD68 reactivity, and extensive localization of myelin debris at sites not co-localizing with CD68 and IBA1 (Fig. 6) or astrocytes (Additional file 3: Fig. S3). This supported impaired uptake of myelin debris and lysosome function in MHV–JHM-infected *Trem2*^−/−^ mice. A magnified view of lesions clearly revealed enhanced reactivity for dMBP in the absence of Trem2, which may be lost in low magnification images assessing demyelination by loss of FM staining. Increased apoptosis in infected *Trem2*^−/−^ mice further suggested impaired removal of dying cells may add to mitigating a remyelinating environment.

MHV–JHM-induced demyelinating lesions contain highly activated IBA1 positive cells. Although microglia rapidly respond to infection, BMDM comprise the largest component of early innate immune cells recruited into the CNS [19]. Interestingly upregulation of *Trem2* mRNA in BMDM correlated directly with the onset of demyelination, while changes in microglia were more subtle compared to naïve cells. This indicates that microglia are constitutively prepared to respond to Trem2 ligands, whereas Trem2 responses by BMDM are directly elicited by sensing damaged tissue and myelin or local metabolites, irrespective of the inflammatory milieu induced by virus or other insults. Moreover, microglia showed no overt change of *Tyrobp* mRNA during acute infection, but dramatic upregulation during demyelination, contrasting the more rapid upregulation of *Tyrobp* mRNA in BMDM. This indicates differential cues for Trem2/Dap12 signaling in microglia and BMDM in the same environment during virus infection. Microglia suffice to uptake myelin in response to MHV–JHM infection as demyelination was evident in CCL2^−/−^ mice, which exhibit significantly impaired CNS recruitment of BMDM [22]. It is also important to note that microglia depletion using PLX5622 either before or during MHV–JHM infection led to sustained clinical disease associated with defective removal of myelin debris without affecting viral control [24]. While no changes in demyelination were observed in the early phase, demyelination remained similar in microglia depleted mice, but decreased in control mice over 2 months [24]. Similar demyelination in the early phase regardless of microglial depletion implicate a contribution of BMDM to this process [24]. However, sustained demyelination in microglia depleted mice emphasize the critical role of microglia for remyelination. One caveat is that CNS infiltrated BMDM exhibited an impaired differentiation phenotype in the absence of microglia [24], potentially altering Trem2 function and phagocytic activity. Although we have not formally assessed a defect in remyelination in our studies, the substantial decline of BMDM within the CNS after day 14 pi support the concept that microglia, rather than BMDM, contribute dominantly to pro-myelinating functions by early removal of myelin debris. This is supported by Syage et al. who demonstrated that a subtype of microglia expressed genes associated with remyelination such as *Igf1* and *Lpl* during MHV mediated demyelination [67]. Our comparative analysis of bulk mRNA expression profiles of microglia and BMDM from MHV–JHM-infected mice also revealed that a subset of genes associated with phagocytosis were impaired in both cell types in the absence of Trem2, including *Lpl* (Figs. 7 and 8). LPL is involved in fatty acid metabolism including scavenging of myelin-derived lipid and Aβ phagocytosis [68–72]. *Lpl* expression is also significantly diminished in the absence of Trem2 in neurogenerative disease and aged mice [7, 9, 73]. Whether Lpl is a downstream mediator of phagocytosis upon Trem2 engagement or can act independently in promoting microglial/BMDM phagocytosis remains to be determined.

## Conclusions

Our data reveal an essential role of Trem2 in the removal of damaged myelin induced by neurotropic MHV–JHM infection. Distinct from both CPZ- and autoimmune-induced demyelination, MHV infection is associated with a highly polarized Th1 response, which controls infectious virus, but during the process induces demyelination. Our results together with findings in other demyelinating models thus highlight that Trem2 dependent regulation of phagocytosis and genes associated with lipid metabolism are similar across demyelination models associated with very distinct inflammatory immune environments, and thus appear to be prominently regulated by sensing damaged tissue and an altered lipid environment. By contrast, functions of Trem2 in regulating antigen presenting cell and T cell functions may be more context-dependent in distinct microbial infections. More research is necessary to characterize the role of Trem2 and LPL specifically in microglia or BMDM in virus-induced demyelination.

## Supplementary Information

**Additional file 1: Figure S1.** Tmem119 is downregulated within the MHV–JHM-induced demyelinated lesions. (A) Representative SC images of Tmem119 Ab staining from WT mice at day 20 pi. White dots indicated the demyelinated lesions. Scale bar = 200 μm. (B) High magnification image of co-staining of Tmem119 and IBA1 with DPAI from (A). Arrow depicts Tmem119^+^IBA1^+^ cells and asterisk depicts IBA1^+^ cells but Tmem119^−^ with activated microglia morphology. Scale bar = 50 μm

**Additional file 2: Figure S2.** Ki67 and active caspase3 positive cells in the SC of naïve WT mice. (A, B) Representative images of Ki67 and active caspase3 positive cells (green) with DAPI (blue) in the ventral funiculus of SC of uninfected mice. Scale bar = 50 μm. (C) Counts of Ki67^+^ and active caspase3^+^ cells per mm^2^. Data show the mean ± SEM from 18 ventral funiculus white matter areas from 3 mice.

**Additional file 3: Figure S3.** Astrocytes do not compensate for impaired uptake of myelin debris by microglia and BMDM. (A) Representative images of GFAP (green), dMBP (red) and DAPI (Blue) in the demyelinated lesion of WT and *Trem2*^−/−^ mice at days 14 and 20 pi. (B) High magnification image from *Trem2*^−/−^ mice (white box in A) revealed no overlapped staining between GFAP and dMBP. Scale bar = 20 μm

**Additional file 4: Figure S4.** Flow cytometry gating strategy for microglia and BMDM purification from SCs. Doublets are excluded by gating on single cells. Staining with Live/Dead cell marker excluded dead cells from the leukocytes gate. Microglia were gated on CD45^int^CD11b^+^ cells and BMDM were gated on CD45^hi^CD11b^+^ Ly6G^−^ cells to exclude Ly6G^+^ neutrophils.

## Data Availability

The data that support the findings in this study are available from the corresponding author (bergmac@ccf.org) upon reasonable request. All Nanostring RNA data (RCC files) were deposited to the NCBI Gene Expression Omnibus databases (GEO) under Accession number GSE206942.

## Abbreviations

Aβ: Amyloid beta
AD: Alzheimer’s disease
APP: Amyloid precursor protein
BMDM: Bone-marrow-derived macrophages
CCL2: CC motif chemokine ligand 2
CCL6: CC motif chemokine ligand 6
CLN: Cervical lymph node
CNS: Central nervous system
CPZ: Cuprizone
Dap12: DNAX activation protein 12
DPBS: Dulbecco’s phosphate-buffered saline
dMBP: Degenerated myelin basic protein
EAE: Experimental autoimmune encephalomyelitis
FACS: Fluorescent activated cell sorting
FC: Fold change
FM: Fluoromyelin
Fn1: Fibronectin 1
GAPDH: Glyceraldehye-3-phosphate dehydrogenase
HEPES: 4-(2-Hydroxyethyl)-1-piperazineethanesulfonic acid
IBA1: Ionized calcium-binding adaptor molecule 1
i.c.: Intracranially
IFN: Interferon
IgG: Immunoglobulin G
IL21R: Interleukin 21 receptor
iNOS: Inducible nitric oxide synthase
ITGAX: Integrin alpha X
LCMV: Lymphocytic choriomeningitis virus
LPL: Lipoprotein lipase
mAb: Monoclonal Ab
MBP: Myelin basic protein
MFI: Mean fluorescent intensity
MHC classII: Major histocompatibility complex class II
MHV–JHM: Glia tropic monoclonal antibody (mAb)-derived 2.2v-1 variant of MHV strain JHM
LOX-1: Lectin-like oxidized low-density lipoprotein 1
PFU: Plaque forming units
pi: Post infection
PRRSV: Porcine reproductive and respiratory syndrome virus
RT-PCR: Real-time polymerase chain reaction
SC: Spinal cord
sTREM2: Soluble TREM2
Thcell: T helper cell
TNF: Tumor necrosis factor
TNFRSF11A: TNF receptor superfamily member 11a
TREM2: Triggering receptor expressed on myeloid cells 2
Viral-N: *Viral-Nucleocapsid*
WT: Wild type
