# Supplementary figures and images for: Trem2 deficiency impairs recovery and phagocytosis and dysregulates myeloid gene expression during virus-induced demyelination
Source: J Neuroinflammation. 2022 Nov 4;19:267. doi: 10.1186/s12974-022-02629-1 (PMC9635103; doi:10.1186/s12974-022-02629-1)

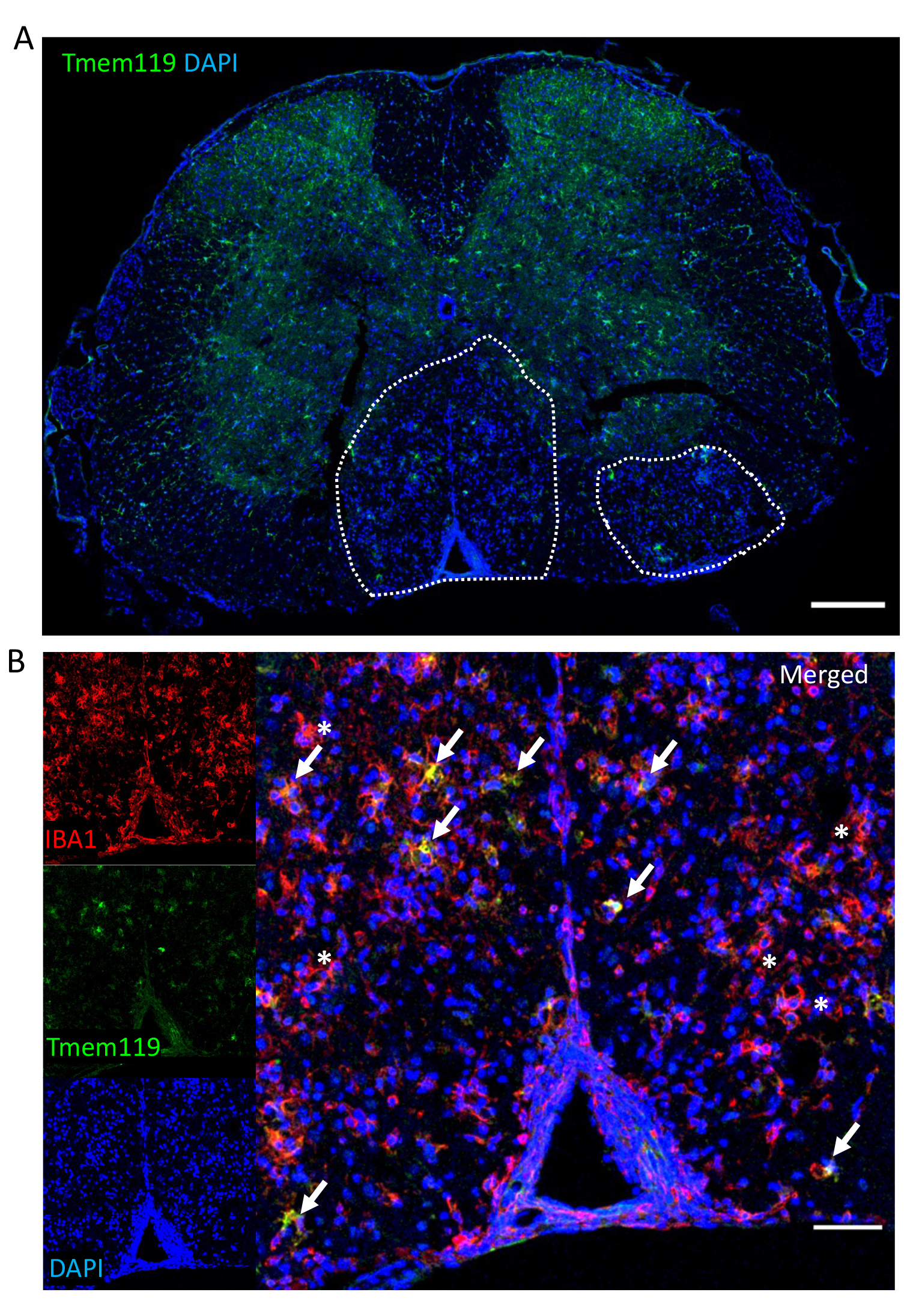

Supplement: Supplementary file 1 — Additional file 1: Figure S1. Tmem119 is downregulated within the MHV–JHM-induced demyelinated lesions. (A) Representative SC images of Tmem119 Ab staining from WT mice at day 20 pi. White dots indicated the demyelinated lesions. Scale bar = 200 μm. (B) High magnification image of co-staining of Tmem119 and IBA1 with DPAI from (A). Arrow depicts Tmem119+IBA1+ cells and asterisk depicts IBA1+ cells but Tmem119− with activated microglia morphology. Scale bar = 50 μm [file 12974_2022_2629_MOESM1_ESM.tif]

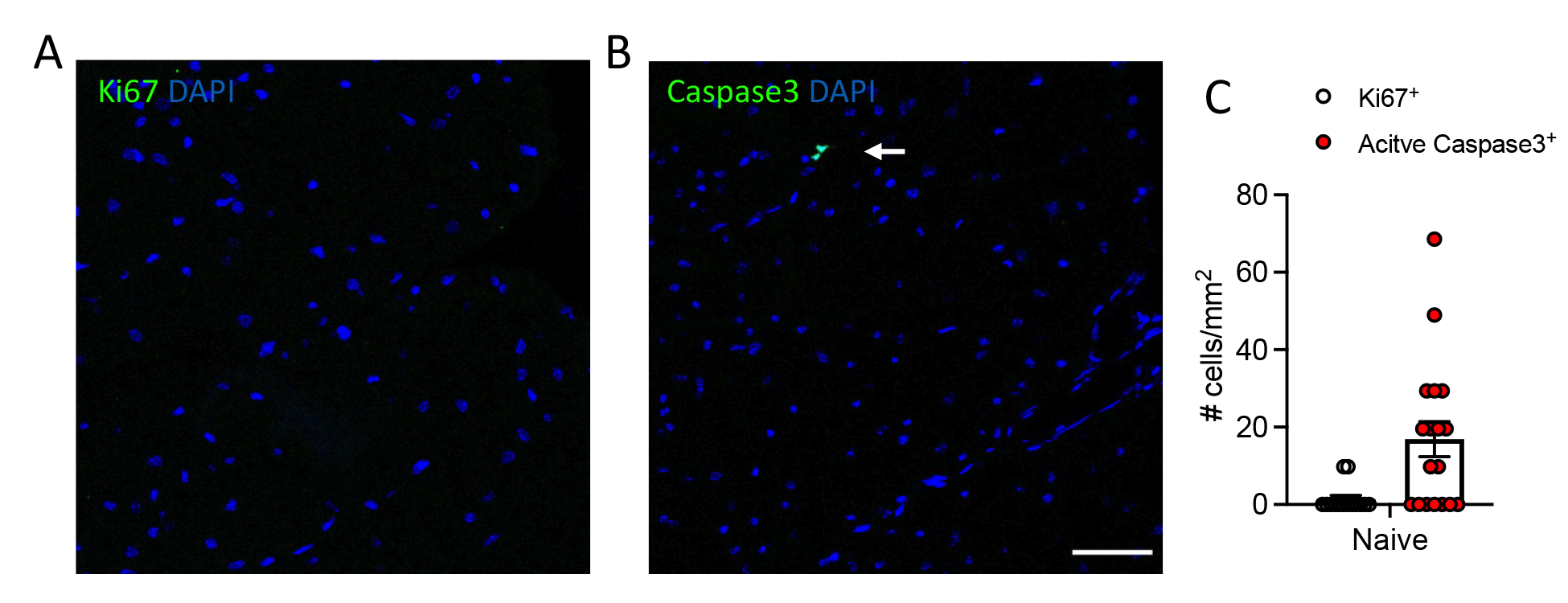

Supplement: Supplementary file 2 — Additional file 2: Figure S2. Ki67 and active caspase3 positive cells in the SC of naïve WT mice. (A, B) Representative images of Ki67 and active caspase3 positive cells (green) with DAPI (blue) in the ventral funiculus of SC of uninfected mice. Scale bar = 50 μm. (C) Counts of Ki67+ and active caspase3+ cells per mm2. Data show the mean ± SEM from 18 ventral funiculus white matter areas from 3 mice. [file 12974_2022_2629_MOESM2_ESM.tif]

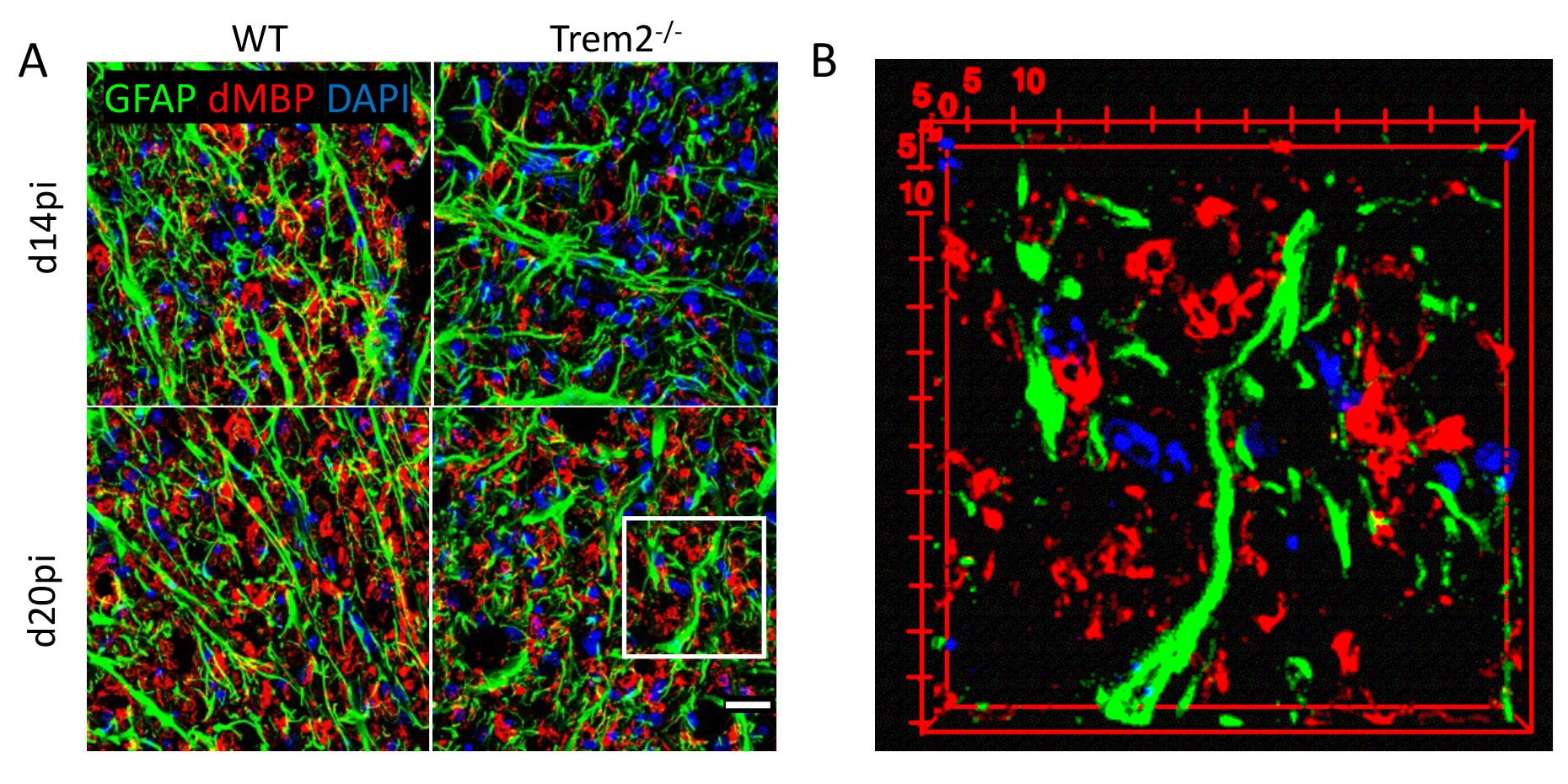

Supplement: Supplementary file 3 — Additional file 3: Figure S3. Astrocytes do not compensate for impaired uptake of myelin debris by microglia and BMDM. (A) Representative images of GFAP (green), dMBP (red) and DAPI (Blue) in the demyelinated lesion of WT and Trem2−/− mice at days 14 and 20 pi. (B) High magnification image from Trem2−/− mice (white box in A) revealed no overlapped staining between GFAP and dMBP. Scale bar = 20 μm [file 12974_2022_2629_MOESM3_ESM.tif]

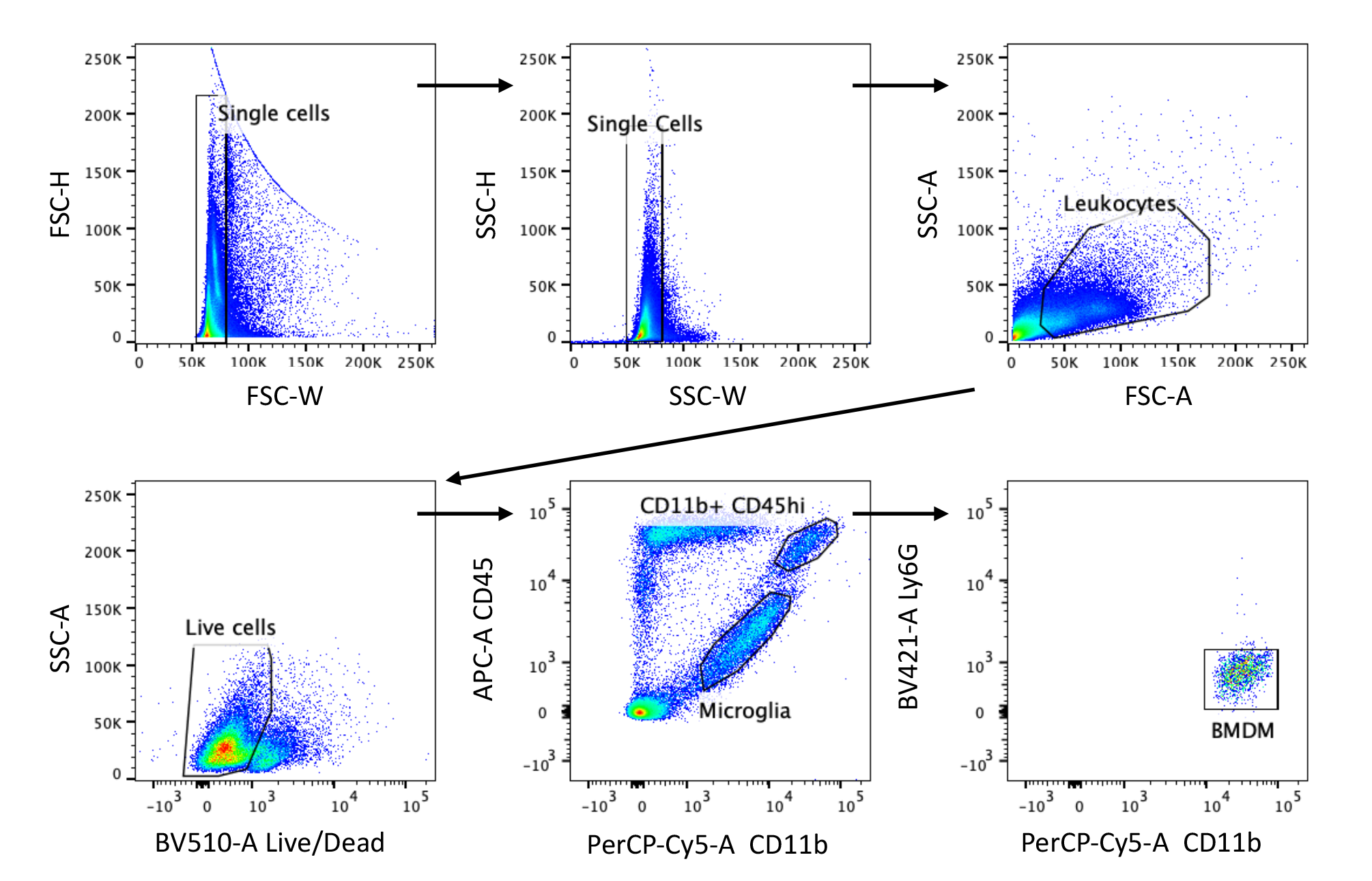

Supplement: Supplementary file 4 — Additional file 4: Figure S4. Flow cytometry gating strategy for microglia and BMDM purification from SCs. Doublets are excluded by gating on single cells. Staining with Live/Dead cell marker excluded dead cells from the leukocytes gate. Microglia were gated on CD45intCD11b+ cells and BMDM were gated on CD45hiCD11b+ Ly6G− cells to exclude Ly6G+ neutrophils. [file 12974_2022_2629_MOESM4_ESM.tif]
